# Long Non-Coding RNA LINC01614 Promotes Aggressive Phenotypes and Ferroptosis Resistance in Papillary Thyroid Cancer Through a Putative miR-4521/IGF2/PI3K-AKT Signaling Axis

**DOI:** 10.3390/genes17101232

**Published:** 2026-10-05

**Authors:** Danielle Quaranto, Nicole R. DeSouza, Michelle Carnazza, Sina Dadafarin, Augustine Moscatello, Codrin Iacob, Humayun K. Islam, Xiu-Min Li, Raj K. Tiwari, Jan Geliebter

**Affiliations:** 1 Department of Pathology, Microbiology, and Immunology, New York Medical College, Valhalla, NY 10595, USA; 2 Department of Research & Development, General Nutraceutical Technology, LLC, Briarcliff Manor, NY 10510, USA; 3 Department of Otolaryngology—Head and Neck Surgery, University of Washington, Seattle, WA 98195, USA; 4 Department of Otolaryngology, New York Medical College, Valhalla, NY 10595, USA; 5 New York Eye and Ear Infirmary of Mount Sinai, New York, NY 10003, USA; 6 Department of Dermatology, New York Medical College, Valhalla, NY 10595, USA

**Keywords:** long non-coding RNAs, ferroptosis, papillary thyroid cancer

## Abstract

**Background/Objectives:** Papillary thyroid cancer (PTC) is the most common endocrine malignancy and is generally associated with favorable clinical outcomes. However, a subset of tumors develops aggressive phenotypes characterized by increased proliferation, migration, therapeutic resistance, and disease progression. Long non-coding RNAs (lncRNAs) have emerged as important regulators of tumor biology, yet the role of Long Intergenic Non-Protein Coding RNA 1614 (LINC01614) in PTC remains poorly understood. This study investigated the functional role of LINC01614 in PTC and its potential involvement in ferroptosis-associated molecular pathways. **Methods:** RNA sequencing data from a repository of 44 paired PTC and adjacent normal thyroid tissue samples were analyzed to identify differentially expressed lncRNAs. LINC01614 expression was evaluated in thyroid cell lines, and CRISPR interference (CRISPRi) was used to generate stable LINC01614 knockdown K1 cell lines. Cellular proliferation, migration, clonogenicity, intracellular iron accumulation, lipid peroxidation, cytokine secretion, and ferroptosis-associated gene expression were assessed using qRT-PCR, RNA sequencing, Western blotting, cytokine array analysis, iron quantification assays, and BODIPY C11 lipid peroxidation imaging. **Results:** LINC01614 was significantly overexpressed in PTC tissues and thyroid cancer cell lines. CRISPRi-mediated repression of LINC01614 reduced cellular proliferation, migration, and clonogenic capacity. Mechanistically, LINC01614 knockdown resulted in an 8.8-fold increase in miR-4521 expression and a 23-fold decrease in IGF2 expression, supporting a potential competing endogenous RNA mechanism. Reduced LINC01614 expression was associated with decreased AKT, GPX4, SLC7A11, and SLC3A2 expression, increased IL-6 secretion, a 3.4-fold increase in intracellular ferrous iron accumulation, and a marked increase in lipid peroxidation following erastin treatment, indicating enhanced sensitivity to ferroptosis. **Conclusions:** These findings identify LINC01614 as a novel regulator of aggressive PTC phenotypes and ferroptosis resistance. The data support a model in which LINC01614 contributes to thyroid cancer progression through modulation of a miR-4521/IGF2/PI3K/AKT signaling axis and downstream anti-ferroptotic pathways. LINC01614 may represent a promising biomarker and potential therapeutic target in papillary thyroid cancer.

## 1. Introduction

Papillary thyroid cancer (PTC) is the most common endocrine malignancy and is generally associated with favorable clinical outcomes and high overall survival rates [1]. Despite its indolent nature, a subset of PTCs acquire aggressive phenotypic characteristics, including enhanced proliferative capacity, migratory potential, therapeutic resistance, and metastatic progression [2]. A major challenge in thyroid cancer research is that aggressive and non-aggressive PTCs frequently arise from many of the same oncogenic driver mutations, including BRAFV600E and RET/PTC rearrangements [3]. While these mutations contribute to tumor initiation and MAPK pathway activation, mutational profiling alone is insufficient to fully explain the molecular heterogeneity and diverse clinical behavior observed among PTC patients [4]. Therefore, the identification of additional molecular mechanisms that contribute to tumor progression is essential for improving prognostic stratification and developing novel therapeutic strategies.

Emerging evidence suggests that epigenetic and transcriptomic dysregulation play central roles in thyroid cancer progression [5]. In particular, long non-coding RNAs (lncRNAs) have gained significant attention due to their ability to regulate gene expression through interactions with DNA, RNA, and protein complexes [6]. These molecules contribute to numerous oncogenic processes including proliferation, migration, invasion, metabolic adaptation, and resistance to cell death [7]. Differential expression analyses have identified several lncRNAs with aberrant expression patterns in thyroid malignancies, suggesting that these molecules may function as critical regulators of tumor behavior and disease progression [8].

Among these, Long Intergenic Non-Protein Coding RNA 1614 (LINC01614) has emerged as a potential oncogenic lncRNA across multiple carcinomas; however, its role in papillary thyroid cancer remains poorly understood [9]. Previous studies have implicated LINC01614 in the regulation of proliferative and metastatic signaling pathways, including PI3K/AKT-mediated oncogenic networks [10]. Additionally, increasing evidence indicates that cancer-associated lncRNAs may influence ferroptosis, an iron-dependent form of regulated cell death characterized by lipid peroxidation and oxidative stress [11]. Ferroptosis has recently been recognized as an important determinant of tumor progression and therapeutic responsiveness in cancer, including thyroid malignancies [12]. However, the relationship between LINC01614 expression and ferroptosis-associated molecular signaling in PTC remains poorly understood.

In the present study, we investigated the functional role of LINC01614 in papillary thyroid cancer using CRISPR interference-mediated knockdown models in K1 thyroid cancer cells. We evaluated the effects of LINC01614 suppression on cellular proliferation, migration, clonogenicity, and ferroptosis-associated molecular pathways. Furthermore, we examined alterations in iron metabolism, lipid peroxidation, and PI3K/AKT-related signaling to better define the mechanistic contribution of LINC01614 to PTC pathogenesis. We hypothesized that LINC01614 promotes aggressive phenotypes and ferroptosis resistance in papillary thyroid cancer through modulation of PI3K/AKT-associated signaling.

## 2. Materials and Methods

### 2.1. Cell Lines

Papillary thyroid cancer (PTC) K1 cells (RRID:CVCL_2537) and immortalized normal thyroid follicular epithelial Nthy-ori-3-1 cells (RRID:CVCL_2659) were cultured in complete RPMI 1640 medium containing 10% fetal bovine serum (FBS), L-glutamine, and phenol red as a pH indicator. Two genetically modified K1 cell lines, K1-LINC01614_1 (K1-L1) and K1-LINC01614_2 (K1-L2), were established through CRISPR interference (CRISPRi)-mediated knockdown, with knockdown subsequently confirmed by quantitative real-time polymerase chain reaction (qRT-PCR). HEK293T cells (CRL-3216), which were used for lentiviral packaging and collection, were cultured in complete Dulbecco’s Modified Eagle Medium (DMEM) containing 10% FBS, 4.5 g/L glucose, L-glutamine, sodium pyruvate, and phenol red pH indicator. All cell lines were maintained at 37 °C with 5% CO2 in a humidified, water-jacketed incubator. For routine passaging, cells were detached using a solution of 0.25% trypsin, 2.21 mM EDTA, and 1X sodium bicarbonate. Standard sterile tissue culture practices were followed for routine maintenance and propagation of all cell lines.

### 2.2. Patient Biobank and Bioinformatic Analysis/Statistics

RNA sequencing data were analyzed from 44 paired papillary thyroid carcinoma (PTC) specimens and corresponding adjacent normal thyroid tissues obtained from the New York Medical College tissue repository. Total RNA was isolated with TRIzol and subsequently purified using the RNeasy Plus Mini Kit (Qiagen, Germantown, MD, USA). RNA quality was evaluated using an Agilent 2100 Bioanalyzer. Whole-transcriptome sequencing was conducted using rRNA-depleted total RNA prepared with the Illumina Ribo-Zero rRNA Removal Kit, following the approach previously described by O’Connell et al. (2021) and Dadafarin et al. (2022) [13,14]. Sequencing reads were mapped to the human reference genome (GRCh38) with STAR (v2.4), and transcript abundance was determined using RSEM (v1.2.14). Differential gene expression was then evaluated using Advaita iPathwayGuide (AdvaitaBio Corporation, Ann Arbor, MI, USA; version not specified).

Statistical comparisons were performed using an unpaired two-tailed Student’s *t*-test. A *p*-value of 0.05 or less was considered statistically significant.

### 2.3. qRT-PCR

Total RNA was isolated from cells grown in 175 cm^2^ tissue culture flasks to approximately 70% confluence using the Zymo Research Quick-RNA Miniprep Kit in accordance with the manufacturer’s instructions. Following extraction, RNA concentration and purity were assessed with an IMPLEN NanoPhotometer Pearl spectrophotometer. For subsequent quantitative real-time polymerase chain reaction (qRT-PCR) analysis, RNA samples were normalized to a final concentration of 50 ng/µL.

Primer sequences ranging from approximately 18–30 base pairs in length were generated using the National Center for Biotechnology Information (NCBI) Primer-BLAST (NCBI, Bethesda, MD, USA; version not specified) tool and subsequently obtained through commercial synthesis.

Primer sequences for LINC01614 were as follows: Forward: 5′-TCAACCAAGAGCGAAGCCAA-3′ and Reverse: 5′-TTGGACACAGACCCTAGCAC-3′. GAPDH was used as the endogenous control with the following primer sequences: Forward: 5′-GTCTCCTCTGACTTCAACAGCG-3′ and Reverse: 5′-ACCACCCTGTTGCTGTAGCCAA-3′. Lyophilized primers were resuspended in nuclease-free H_2_O to prepare 100 µM stock solutions, which were further diluted to 10 µM working solutions for use in qRT-PCR. Each 25 µL reaction contained forward and reverse primers at final concentrations of 0.4 µM each.

Quantitative real-time reverse transcription PCR (one-step RT-qPCR) was performed with qPCRBIO SyGreen 1-Step Go Lo-ROX master mix following the manufacturer’s recommended protocol. Reverse transcription and subsequent PCR amplification were carried out within the same reaction. Reactions were run on a QuantStudio 5 Real-Time PCR instrument equipped with a 96-well format (Applied Biosystems, Thermo Fisher Scientific) (Waltham, MA, USA). The thermal cycling program began with reverse transcription at 45–55 °C for 10 min, followed by enzyme activation and initial denaturation at 95 °C for 2 min. This was followed by 40 amplification cycles, with each cycle consisting of denaturation at 95 °C for 4 s and annealing/extension at 60–65 °C for 20 s. Relative quantification (RQ) of gene expression was calculated using QuantStudio Design & Analysis software (v2.5.1) after normalization to GAPDH. GAPDH served as the endogenous reference because its expression remained stable under the experimental conditions, and it is commonly used for normalization in quantitative RT-PCR analyses of both protein-coding and long non-coding RNA transcripts.

miR-4521 expression was quantified using a miRNA-specific forward primer (5′-GCTAAGGAAGTCCTGTGCTCAG-3′) and a universal reverse primer (5′-TCGTATCCAGTGCAGGGTC-3′). Primers were synthesized by Azenta and used with the qPCRBIO SyGreen 1-Step Go Lo-ROX RT-qPCR kit under the same reaction and cycling conditions described above. Relative miR-4521 expression was determined using QuantStudio Design & Analysis software and normalized to GAPDH expression levels.

### 2.4. CRISPR Interference

CRISPR interference (CRISPRi) was employed to selectively repress LINC01614 transcription using the procedure detailed below.

### 2.5. Preparation of Selection Media and Plates

Luria broth (LB) containing 100 µg/mL ampicillin was used as the selective growth medium and consisted of 10 g/L NaCl, 10 g/L L-tryptone, and 5 g/L yeast extract. For the preparation of solid LB selection plates, 12.5 g of the LB mixture and 10 g of agar were dissolved in 1 L of sterile H_2_O, with ampicillin added to a final concentration of 100 µg/mL.

### 2.6. Single Guide RNA (sgRNA) Selection

Single guide RNAs (sgRNAs) targeting LINC01614 were designed with the Broad Institute CRISPick tool using the human GRCh38 reference genome, SpyoCas9 enzyme settings, and an NGG PAM requirement. The two top-ranked sgRNA candidates were chosen for subsequent CRISPR interference (CRISPRi) experiments. Restriction enzyme-compatible overhangs were incorporated into the sgRNA sequences to enable vector recombination, and the resulting oligonucleotides were commercially synthesized. The oligonucleotide sequences used were as follows:

LINC01614_sg1 (F) 5′-CACCGAAGAGAAAACATACACGCAG-3′,
  (R) 5′-AAACCTGCGTGTATGTTTTCTCTT-3′
 LINC01614_sg2 (F) 5′-CACCGATGTCTACTGCATTTCCTGA-3′;
  (R) 5′-AAACTCAGGAAATGCAGTAGACAT-3′
 Non-specific (NS)_sg (F) 5′-CACCAATCTCGCTTATATAACGAG-3′;
  (R) 5′-AAACCTCGTTATATAAGCGAGATT-3′

Upon arrival, forward and reverse oligonucleotides were reconstituted in 1× TE buffer to generate 100 µM stock solutions. During sgRNA annealing, forward and reverse oligonucleotides were each used at a final concentration of 2.5 µM.

### 2.7. SgRNA Annealing

Forward and reverse sgRNA oligonucleotides were annealed in a 40 µL reaction containing 100 µM forward sgRNA, 100 µM reverse sgRNA, T4 DNA Ligase Buffer, Polynucleotide Kinase (PNK), and molecular-grade nuclease-free water. The reaction was first incubated at 37 °C for 60 min and then heated to 95 °C for 5 min. Following denaturation, the temperature was gradually reduced at a rate of 1 °C per minute until reaching 12 °C to promote annealing of the complementary oligonucleotides.

### 2.8. Restriction Enzyme Plasmid Digestion

The pLV hU6-sgRNA hUbC-dCas9-KRAB-T2a-Puro plasmid (Addgene plasmid #71236), originally obtained from the laboratory of Charles Gersbach, was used for sgRNA cloning and subsequent CRISPR interference (CRISPRi) experiments. For each microgram of plasmid DNA, restriction digestion was performed in a 50 µL reaction containing 5 µL of 10X NEB Buffer 3.1, 1 µL of BsmBI restriction enzyme, and molecular-grade, nuclease-free water. Reactions were incubated at 55 °C for 30 min, after which calf intestinal phosphatase (CIP) was added and incubation was continued at 37 °C for an additional 30 min to minimize vector self-ligation. Digested products were then purified using PCR purification procedures before proceeding to ligation. Each ligation reaction contained 10 µL (500 ng) of the purified dCas9-KRAB-Puro vector that had been digested with BsmBI and treated with CIP.

### 2.9. Plasmid Ligation and Recombination

Purified, restriction-digested plasmid DNA was combined with the previously annealed sgRNA oligonucleotides, T4 DNA Ligase Buffer, and T4 DNA Ligase to generate the recombinant plasmid. Ligation reactions were maintained at room temperature for 2 h to promote incorporation of the sgRNA sequences into the dCas9-KRAB-Puro vector backbone.

### 2.10. Bacterial Transformation of Escherichia coli

NEB 5-alpha competent *E. coli* cells were utilized for transformation and propagation of the recombinant plasmid constructs. Transformation was carried out by heat shocking the competent cells at 42 °C for 30 s, followed by immediate placement on ice. SOC recovery medium was then added, and the transformed cells were allowed to recover at 37 °C with agitation at 250 rpm. Following recovery, the bacterial cells were plated on agar containing ampicillin for selection and incubated overnight at 37 °C.

Individual colonies were subsequently picked and grown overnight in Luria Broth (LB) at 37 °C with shaking at 250 rpm. Recombinant plasmid DNA was recovered from the resulting bacterial cultures using standard plasmid purification methods as described previously. Successful generation of the recombinant vector constructs was confirmed by Sanger sequencing with a U6 promoter sequencing primer (5′-GACTATCATATGCTTACCGT-3′).

### 2.11. Lentivirus Preparation

HEK293T cells served as the producer cell line for lentiviral generation and collection of viral particles. The lentiviral packaging system consisted of the pCMV-VSV-G envelope glycoprotein vector (Addgene #12259), the psPAX2 packaging plasmid (Addgene #12260), and the recombinant pLV hU6-sgRNA hUbC-dCas9-KRAB-T2a-Puro vector carrying the target sgRNA sequence. These plasmids were introduced into HEK293T cells using Opti-MEM reduced-serum medium and TransIT-LT1 transfection reagent in accordance with the manufacturer’s recommended procedure. Following transfection, HEK293T cells were maintained under standard tissue culture conditions to support lentiviral particle generation before viral harvest.

### 2.12. Lentiviral Transduction

Filtered lentiviral particles were introduced into target cells seeded at approximately 30% confluence. For transduction, Polybrene Transfection Reagent (10 mg/mL) was first diluted 1:2.5 with sterile 1X PBS and then further diluted 1:1000 in complete culture medium. Following lentiviral exposure, cells were maintained under standard culture conditions at 37 °C with 5% CO_2_ for 48 h to permit stable integration of the lentiviral construct and expression of the transgene.

### 2.13. Puromycin Selection

The puromycin concentration required for effective selection of transduced target cells had been optimized previously. After lentiviral transduction, the existing culture medium was replaced with complete medium supplemented with 5 µg/mL puromycin to select transduced K1 cells. Cells were maintained at 37 °C with 5% CO_2_ for 48–72 h to allow antibiotic selection to be completed. Following selection, the surviving cell populations were expanded and used in subsequent experiments.

### 2.14. RNA Sequencing

Total RNA was extracted from K1-NS and K1-LINC01614_2 cells in three independent biological replicates using the Zymo Research Quick-RNA Miniprep Kit in accordance with the manufacturer’s instructions. Following purification, RNA samples were stored at −80 °C and subsequently sent to Azenta (Burlington, MA, USA) for next-generation RNA sequencing. Differential gene expression was assessed using the DESeq2 pipeline and the Wald test. Genes meeting both an adjusted *p*-value (Benjamini–Hochberg false discovery rate) of <0.05 and an absolute log_2_ fold change of ≥1 were classified as differentially expressed. The resulting differentially expressed genes (DEGs) were further examined alongside gene ontology and pathway enrichment analyses to characterize molecular and functional changes associated with LINC01614 knockdown.

### 2.15. Trypan Blue Exclusion Assay

Cells were seeded in 6-well tissue culture-treated plates at a density of 30,000 cells per well in 2 mL of complete culture medium. Cell numbers were assessed at 24, 48, 72, and 120 h after seeding. At each time point, cells were detached using 0.25% trypsin and subsequently neutralized with complete culture medium. The resulting cell suspensions were counted using a hemocytometer, and total cell numbers were determined using the following equation:

$$\bar{x\;}\left(\text{average}\;\text{of}\;\text{total}\;\text{cells}\;\text{in}\;\text{all}\;\text{quadrants}\right)*10^{4}*\text{dilution}\;\text{factor}\;\text{number}*mL\;\text{resuspended}\;\text{in}.$$

### 2.16. Colony-Forming Unit Assay

Colony-forming unit (CFU) assays were performed to assess the clonogenic capacity of the cells. Cells were plated at a low density of approximately 200 cells per well in 6-well tissue culture-treated plates and maintained at 37 °C with 5% CO_2_. Cultures were monitored for colony development, with the experimental endpoint defined by maximal colony formation in the K1-NS control wells, corresponding to approximately 50 cells per colony. At the endpoint, colonies were fixed with 100% methanol and subsequently stained with 1% toluidine blue prepared in a 1% borax solution.

### 2.17. Scratch Wound Migration Assay

Cells were plated in 24-well tissue culture-treated plates at a density of 3.0 × 10^4^ cells per well and maintained for 24 h at 37 °C with 5% CO_2_ until a confluent monolayer was established. A linear wound was then created through the center of each well using a sterile 20 µL pipette tip. Wound field measurements were collected immediately after scratch formation (T = 0 h) using brightfield microscopy.

Following initial imaging, cells were returned to standard culture conditions at 37 °C with 5% CO_2_ for an additional 18–24 h to permit migration into the wound area. Wound field measurements were then obtained at the experimental endpoint (T = 18–24 h) using the same imaging parameters. Percent wound healing was determined using the following equation:

Percent Healed (%) = (wound field measurement at T = 18–24 h/wound field measurement at T = 0 h) × 100%.

### 2.18. Western Blot

Cells were grown in tissue culture-treated flasks under standard conditions until reaching approximately 70–90% confluence. After experimental treatment, cells were rinsed with ice-cold 1X phosphate-buffered saline (PBS) and lysed in radioimmunoprecipitation assay (RIPA) buffer containing HALT protease and phosphatase inhibitor cocktail. Lysates were maintained on ice for 15–30 min with periodic vortexing to promote complete protein extraction. Samples were then centrifuged at 12,000× *g* for 15 min at 4 °C, and the clarified supernatants containing total cellular protein were retained for analysis.

Total protein concentrations were measured using a bicinchoninic acid (BCA) protein assay following the manufacturer’s instructions. Equivalent amounts of protein from each sample were mixed with Laemmli sample buffer supplemented with β-mercaptoethanol and heated at 95 °C for 5 min for denaturation. Proteins were resolved by sodium dodecyl sulfate-polyacrylamide gel electrophoresis (SDS-PAGE) on precast polyacrylamide gels and then transferred to polyvinylidene difluoride (PVDF) membranes.

Following protein transfer, membranes were blocked for 1 h at room temperature in 5% bovine serum albumin (BSA) prepared in Tris-buffered saline with Tween-20 (TBST). Primary antibodies were prepared in blocking buffer at the manufacturer-recommended dilutions and incubated with the membranes overnight at 4 °C. After washing with TBST, membranes were incubated with the appropriate horseradish peroxidase (HRP)-conjugated secondary antibodies for 1 h at room temperature. Membranes were washed again with TBST, and immunoreactive bands were detected using enhanced chemiluminescence (ECL) substrate. Images were acquired with the Next Advance Celvin Chemiluminescence Imaging System using SnapProGo software (version not specified). Band intensities were quantified with ImageJ (v1.54), and relative protein expression was determined by normalization to β-actin as the loading control.

### 2.19. Cell Total Iron Quantification Assay

Intracellular iron content was measured using the Cell Total Iron Colorimetric Assay Kit (Invitrogen; catalog no. EEA0009) in accordance with the manufacturer’s recommended procedure. Cells were cultured in tissue culture-treated plates under standard conditions until approximately 70–90% confluence was reached. After experimental treatment, cells were rinsed with ice-cold 1X phosphate-buffered saline (PBS) and harvested by scraping in assay buffer. Cell lysates were generated by mechanical homogenization and subsequently centrifuged to remove cellular debris.

Total cellular iron was determined using a chromogenic detection reagent that produces a colored complex in proportion to the amount of iron present in each sample. Iron standards and experimental cell lysates were added to a 96-well plate in technical replicates following the assay protocol. Absorbance was then recorded with a microplate spectrophotometer at the wavelength specified by the manufacturer.

A calibration curve generated from iron standards of known concentrations was used to determine the iron concentration of each experimental sample. Iron measurements were normalized to either total protein concentration or cell number to enable comparisons among experimental groups.

### 2.20. Cytokine Array

Secreted cytokine profiles were assessed with the RayBio C-Series Human Cytokine Antibody Array C3 (RayBiotech; catalog no. AAH-CYT-3-2) following the manufacturer’s recommended procedure. K1-NS and K1-LINC01614_2 cells were maintained under standard culture conditions until reaching approximately 70–80% confluence. Conditioned culture media were then collected, centrifuged to remove cellular debris, and stored at −80 °C until analysis.

Prior to sample application, array membranes containing pre-spotted capture antibodies against human cytokines were blocked according to the assay protocol. Membranes were then incubated with conditioned media, washed, and exposed to biotin-conjugated detection antibodies. Horseradish peroxidase (HRP)-linked streptavidin was subsequently applied, followed by the appropriate chemiluminescent detection reagents as specified by the manufacturer.

Chemiluminescent signals were captured using the Next Advance Celvin Chemiluminescence Imaging System with SnapProGo software. Cytokine abundance was evaluated by densitometric quantification of individual spot intensities using ImageJ. Signal values were normalized against the positive control reference spots included on each membrane, and differences in cytokine expression between the experimental groups were evaluated for subsequent molecular characterization.

### 2.21. Lipid Peroxidation Assay with Erastin Treatment

Lipid peroxidation was assessed by fluorescence imaging with BODIPY™ 581/591 C11 followed by quantitative analysis in ImageJ. Cells were plated on sterile coverslips in 24-well tissue culture-treated plates and exposed to erastin (MedChemExpress (Monmouth Junction, NJ, USA); catalog no. 571203-78-6) to induce ferroptosis. A 10 mM erastin stock solution was diluted in complete culture medium to a final concentration of 10 µM, and 500 µL of the resulting treatment medium was added to each well. Following erastin exposure, cells were incubated with 5 µM BODIPY™ 581/591 C11 in serum-free, phenol red-free medium for 30–35 min at 37 °C while protected from light. Cells were then washed with Hank’s Balanced Salt Solution (HBSS) and mounted with ProLong™ Gold Antifade Mountant containing DAPI. Fluorescence images were acquired using a Nikon Eclipse Ti microscope with NIS-Elements imaging software (v5).

Oxidized lipid species were visualized in the green fluorescence channel, whereas reduced lipid species were detected in the red fluorescence channel. Fluorescence intensities were subsequently quantified using ImageJ software.

## 3. Results

### 3.1. RNA Sequencing and Bioinformatic Analysis of Patient Data

RNA sequencing data from 44 paired papillary thyroid cancer (PTC) and adjacent normal thyroid tissue samples obtained from the New York Medical College tissue repository were analyzed to assess differential expression of protein-coding and non-coding transcripts. Analysis using Advaita’s iPathwayGuide identified more than 450 differentially expressed non-coding genes. LINC01614 was among the most significantly upregulated long non-coding RNAs (lncRNAs), demonstrating an approximately 8-fold increase in PTC relative to matched adjacent normal thyroid tissue (log_2_ fold change ≈ 3; Figure 1). Based on its marked differential expression, LINC01614 was selected for further molecular characterization to investigate its potential contribution to PTC pathogenesis.

### 3.2. Generation of In Vitro Cell Model Using CRISPR Interference (CRISPRi)

To examine the functional significance of LINC01614 in papillary thyroid cancer, CRISPR interference (CRISPRi) was used to establish K1 cell lines with transcriptional repression of LINC01614. Initial qRT-PCR analysis demonstrated that LINC01614 expression was approximately 50% greater in K1 papillary thyroid cancer cells than in immortalized normal thyroid epithelial Nthy-ori-3-1 cells (Figure 2a). Two independent LINC01614-targeting CRISPRi cell lines, K1-LINC01614_1 (K1-L1) and K1-LINC01614_2 (K1-L2), were subsequently generated in K1 cells and compared with a non-specific CRISPRi control (K1-NS). Successful repression of LINC01614 was confirmed by qRT-PCR in both knockdown cell lines. Compared with K1-NS cells, LINC01614 expression decreased by approximately 75% in K1-LINC01614_1 and 60% in K1-LINC01614_2 cells (Figure 2b). K1-LINC01614_2 demonstrated greater consistency and reproducibility of LINC01614 repression across biological replicates and was therefore chosen for subsequent RNA sequencing analysis.

To further evaluate the molecular consequences associated with LINC01614 suppression, RNA sequencing analysis was performed on K1-LINC01614_2 cells and compared to K1-NS controls. Transcriptomic analysis identified numerous differentially expressed genes (DEGs), many of which have previously been implicated in cancer-associated biological processes, including proliferation, migration, stress adaptation, metabolic regulation, and resistance to regulated cell death pathways (Figure 3).

The substantial changes in gene expression observed following LINC01614 repression led us to examine the functional effects of LINC01614 knockdown on cellular sustainability and tumor-associated phenotypes. Cell proliferation assays demonstrated that transcriptional repression of LINC01614 decreased the proliferative capacity of K1 papillary thyroid cancer cells by approximately 45% over a 120 h period (Figure 4a). Based on this pronounced reduction in proliferation, we subsequently investigated whether LINC01614 repression also affected the ability of K1 cells to maintain long-term growth and self-renewal.

Clonogenic colony formation assays showed that LINC01614 repression resulted in an approximately 34% decrease in the clonogenic capacity of K1 cells relative to non-specific control cells (Figure 4b,c). Together, these results indicate that LINC01614 supports both proliferative and self-sustaining properties of papillary thyroid cancer cells.

To assess the influence of LINC01614 on cellular phenotypes associated with metastatic potential in vitro, cell migration was evaluated using a scratch wound assay. Both LINC01614 knockdown cell lines exhibited an approximately 50% reduction in migration relative to the non-specific control cells (Figure 5). These findings indicate that transcriptional repression of LINC01614 impairs the migratory capacity of K1 cells in vitro, suggesting a potential role for LINC01614 in promoting metastatic-associated cellular behavior.

### 3.3. CRISPRi-LINC01614 Cell Model Leads to Transcriptional Changes Associated with Ferroptosis

To further characterize the molecular pathways associated with LINC01614-mediated tumor progression, we examined processes involved in ferroptosis and the regulation of oxidative stress. RNA sequencing and gene ontology analyses identified several differentially expressed genes associated with lipid metabolism, cellular redox balance, and iron-dependent stress responses following CRISPRi-mediated repression of LINC01614. A selection of the top differentially expressed genes is provided in Table A1. These transcriptional changes led us to investigate whether LINC01614 contributes to an anti-ferroptotic phenotype in papillary thyroid cancer cells.

RNA sequencing demonstrated marked decreases in the expression of SLC7A11 and SLC3A2, which encode the two protein components of System xCT, the cystine/glutamate antiporter, following LINC01614 repression [15]. SLC7A11, the catalytic subunit of System xCT, showed an approximately 80% reduction in expression, while SLC3A2, the associated heavy-chain subunit, was reduced by approximately 75% compared with K1-NS control cells (Figure 6). Together, these findings indicate that LINC01614 is associated with the maintenance of the System xCT pathway and suggest a potential role for LINC01614 in supporting ferroptosis resistance in papillary thyroid cancer cells.

To further investigate ferroptosis-associated pathways, we evaluated GPX4 expression by Western blot analysis. GPX4 is a key antioxidant defense that prevents ferroptotic cell death downstream of the xCT system [16]. Thus, changes in GPX4 expression would alter cell susceptibility to ferroptotic cell death.

Western blot analysis showed reduced GPX4 protein expression following LINC01614 repression. Relative to K1-NS cells, normalized GPX4 expression was significantly reduced in both K1-LINC01614_1 and K1-LINC01614_2 cells, with the greatest reduction observed in K1-LINC01614_2 cells (Figure 7). Parental K1 cells were included in the representative blot as a reference but were not included in the densitometric or statistical analysis.

As alterations in ferroptosis-associated gene expression became evident following LINC01614 repression, we next sought to determine whether suppression of LINC01614 increased the susceptibility of papillary thyroid cancer cells to ferroptosis-related processes. Given the established relationship between oxidative stress, inflammatory signaling, and ferroptotic regulation, we first evaluated the cytokine expression landscape of K1-LINC01614 knockdown cells to identify signaling pathways potentially associated with ferroptosis sensitivity. Of note, interleukin-6 (IL-6) is associated with signaling pathways that can influence cellular oxidative stress and ferroptosis susceptibility, guiding us to analyze cytokine array levels [17].

Human cytokine array analysis revealed about a 75% increase in inflammation-associated cytokine IL-6 secretion from LINC01614 knockdown cells compared to K1-NS controls (Figure 8).

We next sought to evaluate intracellular iron levels within our experimental cell lines to determine whether the intracellular labile iron pool differed following repression of LINC01614. Since ferroptosis is an iron-dependent form of regulated cell death, increased intracellular iron accumulation would be expected to enhance cellular susceptibility to ferroptotic signaling and oxidative lipid damage [11]. Therefore, quantification of intracellular iron was performed to assess whether LINC01614 knockdown altered iron homeostasis in a manner consistent with increased ferroptosis sensitivity.

Quantification of intracellular iron revealed that repression of LINC01614 significantly increased intracellular Fe^2+^ accumulation. K1-LINC01614_1 cells exhibited an approximately 3.4-fold increase in intracellular iron levels relative to K1-NS control cells (Figure 9), supporting a role for LINC01614 in regulating iron homeostasis and ferroptosis sensitivity.

In addition to changes in intracellular iron accumulation, lipid peroxidation of cellular membranes represents a key feature of ferroptotic cell death. We therefore examined whether transcriptional repression of LINC01614 increased susceptibility to lipid peroxidation following pharmacological induction of ferroptosis with Erastin [11].

Following erastin treatment, BODIPY™ 581/591 C11 staining revealed a substantial increase in lipid peroxidation in LINC01614 knockdown cells [18]. Oxidized lipid fluorescence increased approximately 5.6-fold and 6.4-fold in K1-LINC01614_1 and K1-LINC01614_2 cells, respectively, compared to K1-NS controls (Figure 10). Conversely, non-oxidized lipid fluorescence was reduced to approximately 0.27-fold of control levels in both knockdown cell lines. These findings demonstrate increased lipid peroxidation following LINC01614 repression under ferroptosis-inducing conditions.

### 3.4. In Silico Prediction of LINC01614 Mechanism of Action in PTC

Competing endogenous RNA (ceRNA) networks represent a well-established mechanism through which long non-coding RNAs regulate gene expression [17]. As a long non-coding RNA exceeding 2000 nucleotides in length, LINC01614 possesses substantial potential for interactions with microRNAs (miRNAs) and other regulatory RNA species. Identification of the molecular mechanisms through which LINC01614 exerts its oncogenic effects may therefore provide important insight into the pathways governing papillary thyroid cancer progression and ferroptosis-associated signaling.

Given the emerging role of lncRNAs as molecular sponges within ceRNA regulatory networks, in silico analysis of potential miRNA binding partners was performed to further investigate the mechanistic role of LINC01614. Identification of candidate miRNA interactions may help elucidate how LINC01614 contributes to oncogenic signaling, oxidative stress regulation, and ferroptosis resistance while also revealing additional molecular targets with potential therapeutic relevance in papillary thyroid cancer.

To begin the in silico analysis, ENCORI and miRcode software were used to search for potential miRNA binding partners of LINC01614. A list of about 19 miRNAs was generated and ranked based on predicted and actual binding evidence. Of the 19 miRNAs, miR-4521 was predicted to have the strongest interaction with LINC01614 and confirmed using IntaRNA software (v3.4.1). Expression of miR-4521 was validated in our cell lines using qRT-PCR. Analysis revealed a significant increase in miR-4521 expression following repression of LINC01614. Compared to K1-NS control cells, LINC01614 knockdown cells exhibited an approximately 8.8-fold increase in miR-4521 expression (Figure 11), suggesting that LINC01614 may participate in a potential competing endogenous RNA (ceRNA) regulatory network involving miR-4521.

Upon qRT-PCR confirmation of increased miR-4521 expression following CRISPRi-mediated repression of LINC01614, we next sought to investigate downstream target mRNAs associated with this regulatory interaction utilizing the same procedure as above. Previous studies have identified insulin-like growth factor 2 (IGF2) as a validated target of miR-4521 and a known activator of the PI3K/AKT signaling pathway [19]. RNA sequencing analysis revealed decreased IGF2 expression in K1-LINC01614 knockdown cells compared to K1-NS control cells, and this was confirmed using qRT-PCR. Analysis revealed a marked reduction in IGF2 expression following repression of LINC01614. Compared to K1-NS control cells, LINC01614 knockdown cells exhibited an approximately 23-fold decrease, corresponding to a 96% reduction in IGF2 expression (Figure 12). This inverse relationship with increased miR-4521 expression supports the proposed LINC01614/miR-4521/IGF2 regulatory axis.

These findings are consistent with a competing endogenous RNA (ceRNA) mechanism in which repression of LINC01614 increases the availability of miR-4521, thereby enhancing suppression of its downstream target mRNA, IGF2. Given the established role of IGF2 in promoting proliferative and pro-survival signaling through PI3K/AKT activation, these results further support the hypothesis that LINC01614 contributes to papillary thyroid cancer progression through modulation of oncogenic signaling networks.

Based on these findings, we hypothesized a potential mechanism through which LINC01614 contributes to papillary thyroid cancer progression via regulation of the PI3K/AKT pathway and ferroptosis-associated signaling. The PI3K/AKT pathway is a central regulator of cellular growth, proliferation, metabolism, and protein synthesis and has additionally been implicated in the regulation of ferroptosis-associated genes, including SLC7A11 and GPX4 [19]. Literature review and transcriptomic analysis collectively suggested that LINC01614 may influence these pathways through a competing endogenous RNA (ceRNA)-mediated mechanism involving miR-4521 and IGF2.

To determine whether the proposed LINC01614-miR-4521-IGF2 axis influences downstream signaling, we evaluated AKT protein expression by Western blot analysis.

IGF2 is an upstream activator of PI3K/AKT signaling, which regulates downstream processes including protein synthesis, cellular growth, and proliferation. To investigate whether LINC01614 repression was associated with changes in this pathway, we evaluated total AKT protein expression by Western blot analysis. Because AKT phosphorylation was not measured, this analysis did not directly assess pathway activation.

Western blot analysis showed that LINC01614 repression was associated with reduced total AKT protein expression. Relative to K1-NS cells, K1-LINC01614_1 cells showed an approximately 90% reduction in normalized AKT abundance (Figure 13). These findings are consistent with possible modulation of PI3K/AKT signaling by LINC01614; however, pathway activation was not directly evaluated.

This finding aligns with the observed decrease in IGF2 expression and increase in miR-4521 levels. Together, these findings are consistent with a potential miRNA-mediated regulatory mechanism involving LINC01614. However, additional mechanistic and rescue experiments will be required to determine whether the observed changes in miR-4521 and IGF2 directly mediate the effects of LINC01614 repression on PI3K/AKT signaling and ferroptosis susceptibility.

Under normal physiological conditions, lower expression levels of LINC01614 would permit increased availability of miR-4521, thereby enhancing suppression of IGF2 expression and maintaining baseline activation of PI3K/AKT signaling. Consequently, expression of anti-ferroptotic genes such as SLC7A11 and GPX4 would remain at physiologic levels, allowing cells to retain sensitivity to ferroptosis-associated oxidative stress.

In papillary thyroid cancer, however, increased expression of LINC01614 may functionally sponge miR-4521, reducing its inhibitory effects on IGF2 and resulting in elevated IGF2 expression. Increased IGF2-mediated activation of the PI3K/AKT pathway would subsequently enhance downstream proliferative and pro-survival signaling while promoting increased expression of anti-ferroptotic genes. Collectively, this mechanism would support tumor growth, enhanced proliferative capacity, and decreased sensitivity to ferroptotic cell death in papillary thyroid cancer cells.

## 4. Discussion

Papillary thyroid cancer (PTC) is typically associated with a favorable prognosis, although a subset of tumors develop more aggressive characteristics, including enhanced proliferation and migration, resistance to therapy, and increased metastatic potential. Established oncogenic drivers, including BRAFV600E, play important roles in PTC development but do not completely account for the molecular and phenotypic variability observed across patients. Consequently, further characterization of the molecular factors that contribute to aggressive PTC behavior remains important. In this study, we identified the long non-coding RNA LINC01614 as significantly overexpressed in PTC and showed that it contributes to cellular phenotypes involving proliferation, clonogenic growth, migration, and resistance to ferroptosis [20,21].

Functional repression of LINC01614 using CRISPR interference significantly reduced proliferation, clonogenicity, and migratory capacity in K1 papillary thyroid cancer cells. These findings support previous studies describing LINC01614 as an oncogenic lncRNA in multiple malignancies while additionally establishing a previously uncharacterized role in papillary thyroid cancer. RNA sequencing analysis further demonstrated that repression of LINC01614 broadly altered gene expression programs associated with tumor progression, stress adaptation, and metabolic regulation. Together, these findings suggest that LINC01614 contributes to the maintenance of an aggressive tumor phenotype through widespread transcriptomic regulation [11,12].

A major finding of this study was the relationship between LINC01614 expression and pathways involved in ferroptosis. Ferroptosis is a regulated, iron-dependent form of cell death distinguished by intracellular iron accumulation and oxidative damage to membrane lipids. Although ferroptosis has gained attention as a potential therapeutic vulnerability in cancer, the involvement of long non-coding RNAs in regulating this process in papillary thyroid cancer remains incompletely characterized. In our study, transcriptional repression of LINC01614 was accompanied by reduced expression of the anti-ferroptotic factors SLC7A11, SLC3A2, and GPX4, together with increased intracellular iron levels and enhanced lipid peroxidation following erastin exposure. Collectively, these findings are consistent with a potential role for LINC01614 in supporting ferroptosis resistance in PTC cells.

Interestingly, cytokine array analysis also revealed increased IL-6 expression following LINC01614 repression. IL-6 has previously been implicated in the regulation of iron metabolism, reactive oxygen species (ROS) production, and ferroptosis-associated inflammatory signaling [22,23]. While the precise contribution of IL-6 within this system remains unclear, its increased expression may represent a compensatory cellular response to oxidative stress and ferroptosis sensitization. Further mechanistic studies will be required to determine whether IL-6 directly contributes to the ferroptotic phenotype observed following repression of LINC01614. Collectively, these transcriptomic findings suggest that repression of LINC01614 is associated with broad alterations in gene expression in papillary thyroid cancer cells. Although these results are promising, additional mechanistic studies are warranted to determine how LINC01614 regulates these transcriptional networks and contributes to papillary thyroid cancer progression.

Our findings support a potential competing endogenous RNA (ceRNA) regulatory mechanism involving LINC01614, miR-4521, and IGF2 (Figure 14). Bioinformatic analyses identified miR-4521 as a predicted binding partner of LINC01614, while repression of LINC01614 was associated with increased miR-4521 expression and a corresponding decrease in IGF2 expression. These observations are consistent with a model in which reduced LINC01614 expression increases the availability of miR-4521, potentially enhancing miR-4521-mediated suppression of its predicted downstream target, IGF2. However, direct molecular interactions among LINC01614, miR-4521, and IGF2 were not experimentally validated in the present study.

IGF2 is an established upstream regulator of PI3K/AKT signaling, a pathway involved in cellular proliferation, survival, and the regulation of ferroptosis-associated factors, including SLC7A11 and GPX4 [24]. Consistent with the proposed regulatory relationship, LINC01614 repression was associated with reduced total AKT protein abundance, with the strongest reduction observed in K1-LINC01614_1 cells. These findings suggest that LINC01614 may influence PI3K/AKT-associated signaling in PTC and are consistent with the observed reduction in IGF2 expression and increase in miR-4521 levels. However, because AKT phosphorylation was not assessed and the Western blot analysis included two biological replicates, these findings should be considered preliminary and do not establish altered pathway activation. Further studies evaluating phosphorylated AKT, downstream pathway targets, and rescue experiments are required to confirm this proposed mechanism.

Several limitations should be taken into consideration when interpreting these findings. The present study was conducted primarily using a single papillary thyroid cancer cell line; therefore, validation in additional PTC cell lines and experimental models will be important to determine whether the observed effects of LINC01614 are broadly applicable across PTC.

Additional considerations relate to the use of CRISPR interference (CRISPRi) for transcriptional repression of LINC01614. The sgRNAs employed in this study were designed using the Broad Institute CRISPick platform to prioritize predicted on-target activity while limiting potential off-target effects. Moreover, two independently designed sgRNAs targeting the LINC01614 promoter achieved comparable levels of repression and produced consistent effects on proliferation, clonogenicity, and migration, supporting the reproducibility of the observed phenotypes. However, CRISPRi relies on dCas9-KRAB-mediated transcriptional repression and associated changes in local chromatin. As a result, contributions from altered chromatin structure, disruption of nearby regulatory elements, or changes in the expression of neighboring transcripts cannot be entirely excluded. Future studies incorporating orthogonal gene-silencing strategies, assessment of neighboring transcript expression, and systematic evaluation of potential off-target effects will be important for further establishing the specificity of LINC01614-associated phenotypes in papillary thyroid cancer.

In addition, although bioinformatic predictions and expression analyses support a potential LINC01614–miR-4521–IGF2 regulatory axis, direct experimental validation of this proposed mechanism was outside the scope of the current study. Rescue experiments were also not conducted. Therefore, the observed increase in miR-4521 expression, reduction in IGF2 expression, reduced total AKT abundance, and increased susceptibility to ferroptotic stress following LINC01614 repression demonstrate associations but do not establish a direct causal relationship among these molecular events. Future studies incorporating luciferase reporter assays, AGO2 RNA immunoprecipitation, RNA pull-down experiments, miR-4521 inhibition, IGF2 overexpression, and complementary rescue approaches will be important for confirming the predicted molecular interactions and determining the functional relevance of the proposed LINC01614–miR-4521–IGF2 regulatory axis.

Similarly, several complementary findings, including increased intracellular iron accumulation, enhanced lipid peroxidation following erastin exposure, and decreased expression of the ferroptosis-associated proteins GPX4 and SLC7A11, are consistent with increased ferroptotic susceptibility following LINC01614 repression. However, direct confirmation that these phenotypic changes are dependent on ferroptosis was not established in the present study. Furthermore, PI3K/AKT pathway activity was evaluated based on total AKT protein expression rather than phosphorylation-specific measurements of AKT activation. Future investigations should therefore incorporate ferroptosis rescue experiments using inhibitors such as ferrostatin-1 or liproxstatin-1, GPX4 or SLC7A11 overexpression, phosphorylation-specific AKT analysis, and additional markers of ferroptosis. These approaches will help determine whether the observed cellular effects are directly dependent on ferroptotic mechanisms and further clarify the relationship among LINC01614 expression, PI3K/AKT signaling, and ferroptosis.

## 5. Conclusions

In conclusion, this study identifies LINC01614 as a potential oncogenic long non-coding RNA involved in papillary thyroid cancer progression and resistance to ferroptotic stress. CRISPRi-mediated repression of LINC01614 resulted in significant reductions in cellular proliferation, clonogenic growth, and migration, accompanied by increased intracellular iron accumulation, enhanced lipid peroxidation, and greater sensitivity to erastin-induced ferroptotic stress. At the molecular level, our findings are consistent with a potential ceRNA regulatory network involving LINC01614, miR-4521, and IGF2, which may contribute to modulation of PI3K/AKT signaling and downstream anti-ferroptotic factors. Together, these results support a role for LINC01614 in maintaining aggressive tumor-associated properties and cellular adaptation to oxidative stress in papillary thyroid cancer. Further mechanistic investigation of this pathway may improve our understanding of therapeutic vulnerabilities in PTC and potentially inform the development of targeted strategies for more aggressive forms of thyroid cancer.

## Figures and Tables

**Figure 1:**
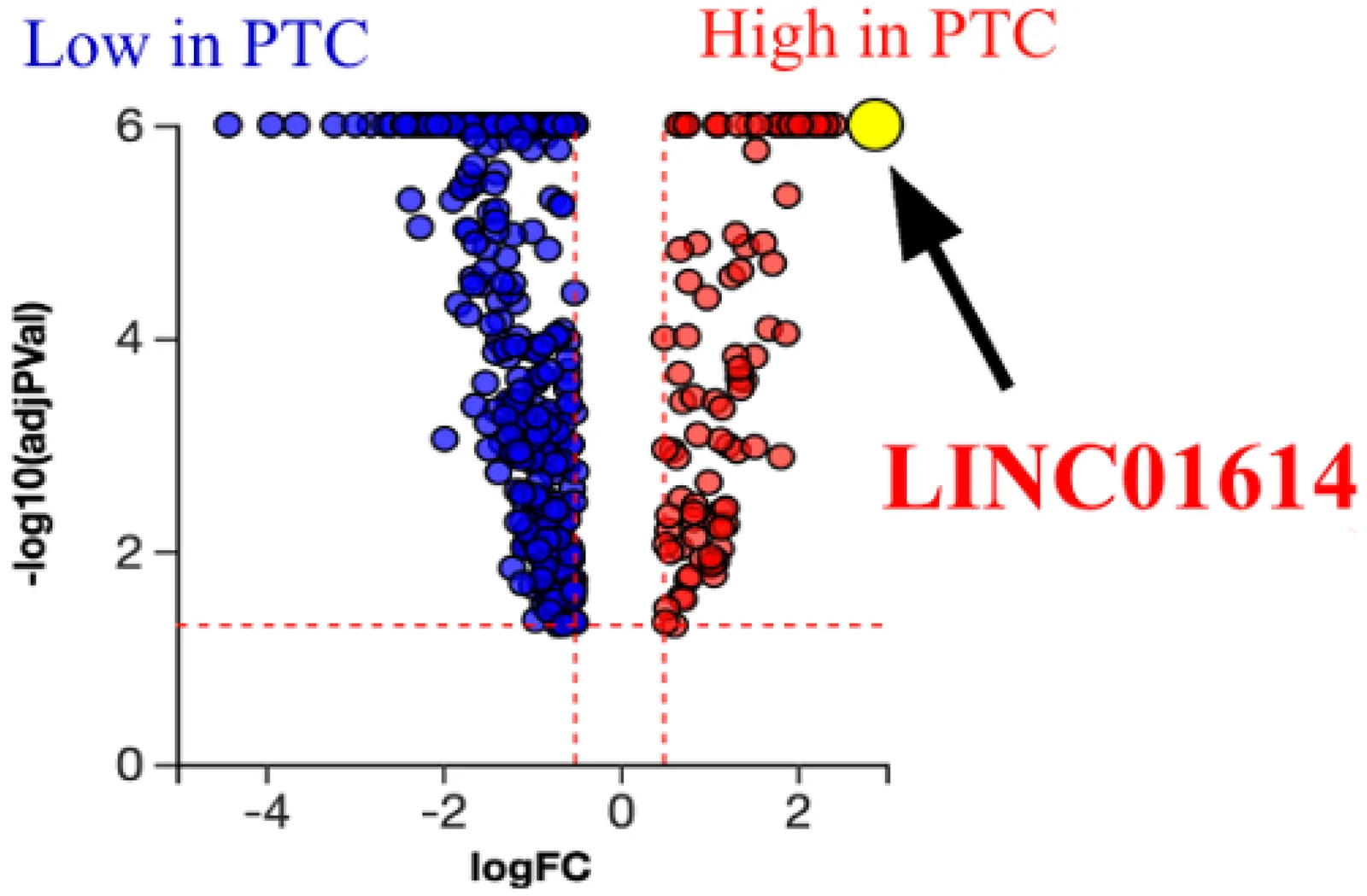
RNA sequencing identifies LINC01614 as a highly upregulated non-coding transcript in PTC. Volcano plot depicting approximately 450 differentially expressed non-coding genes in paired PTC and adjacent normal thyroid tissue samples from the NYMC tissue repository. LINC01614 was among the most highly upregulated non-coding transcripts, exhibiting an approximately 8-fold increase in PTC relative to matched adjacent normal tissue (log_2_ fold change ≈ 3).

**Figure 2:**
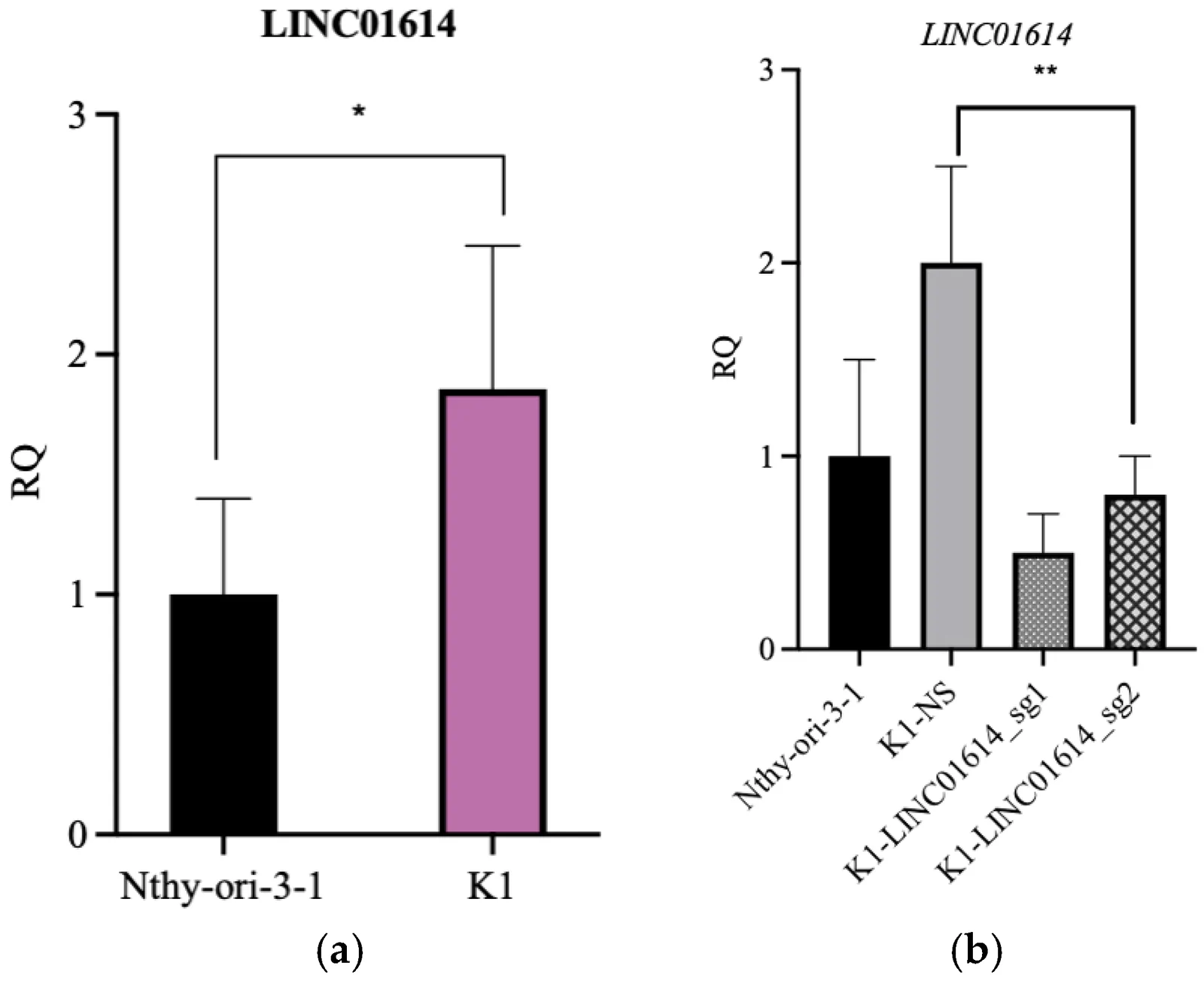
LINC01614 expression is elevated in the PTC K1 cell line and reduced following CRISPRi-mediated repression. (**a**) Comparison of LINC01614 expression between the PTC K1 cell line and the immortalized normal thyroid follicular epithelial Nthy-ori-3-1 cell line. (**b**) qRT-PCR confirmation of LINC01614 repression in two independently generated CRISPRi cell lines, K1-LINC01614_1 and K1-LINC01614_2, compared with the K1-NS control. Statistical significance was assessed using an unpaired *t*-test; *n* = 3 per group (3 technical replicates per experiment), * *p* ≤ 0.05; ** *p* ≤ 0.01.

**Figure 3:**
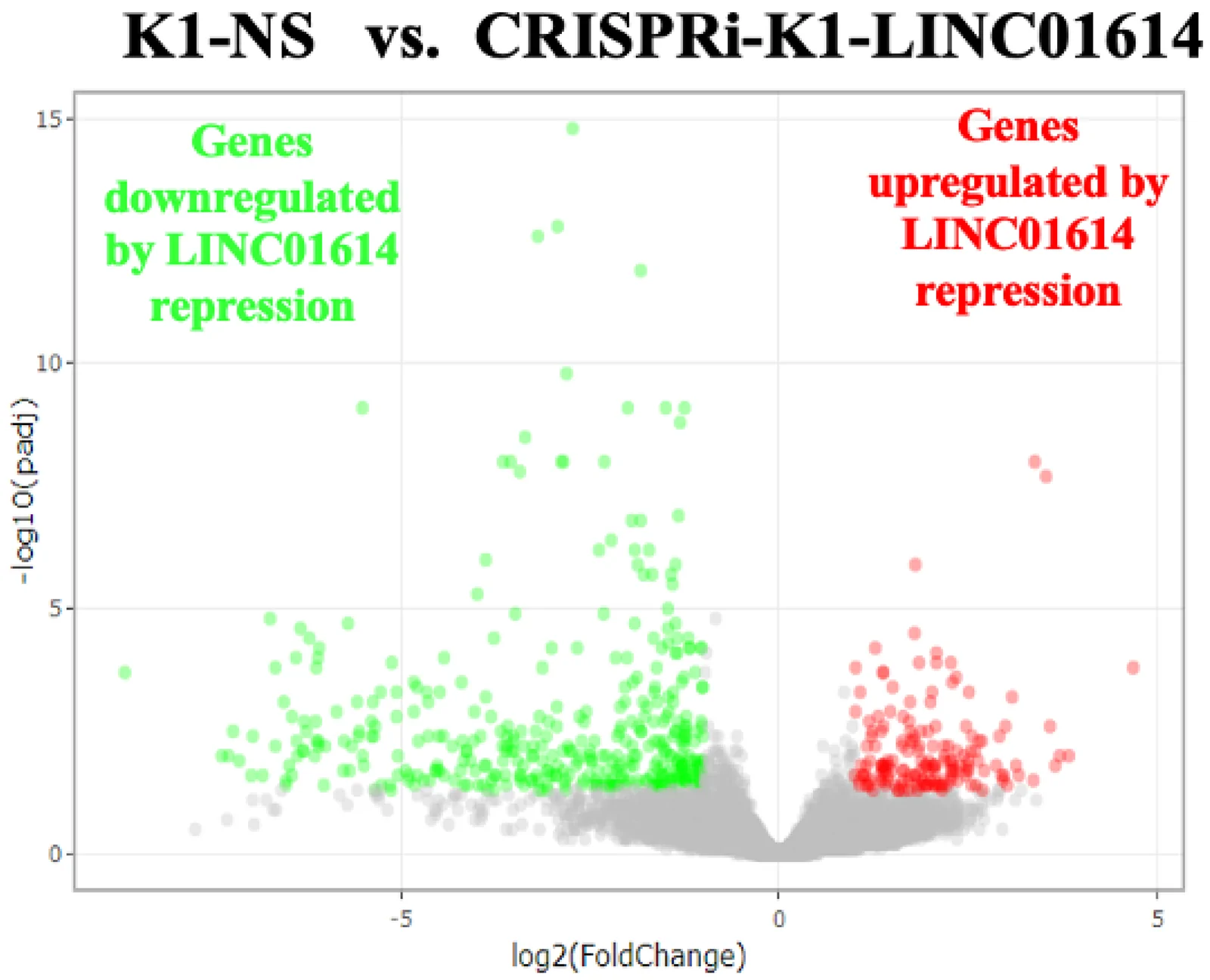
RNA-seq analysis identifies widespread gene expression changes following transcriptional repression of LINC01614 in K1 cells. Volcano plot illustrating differentially expressed genes (DEGs) between K1-NS and K1-LINC01614_2 cells. Genes exhibiting increased expression in K1-LINC01614_2 relative to K1-NS are shown in red, whereas genes with decreased expression are shown in green. Gray area represents nonsignificant down- and upregulated genes. Differential expression analysis was performed using DESeq2 (Azenta) with the Wald test. DEGs were defined by an adjusted *p*-value < 0.05 and an absolute log_2_ fold change ≥ 1.

**Figure 4:**
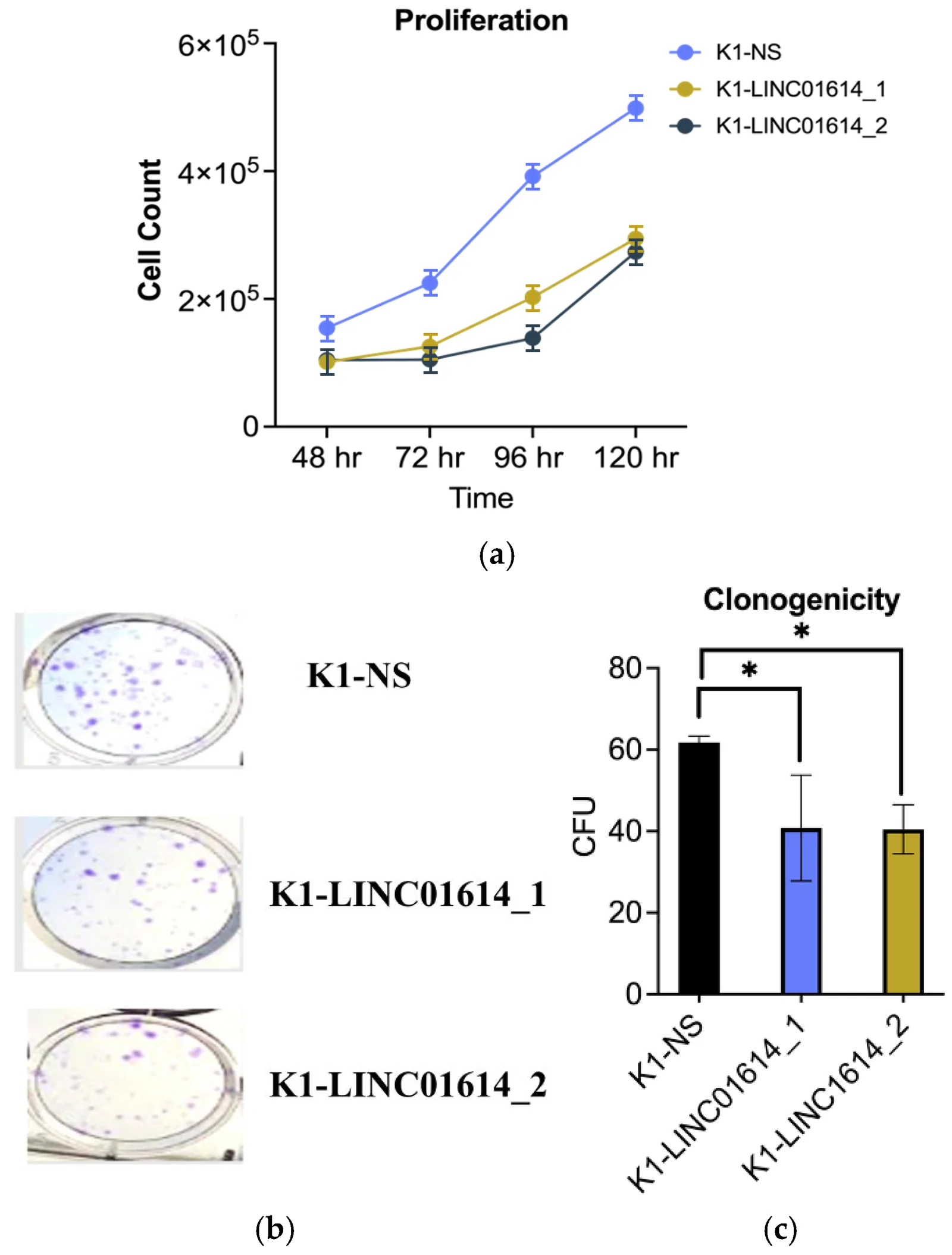
Transcriptional repression of LINC01614 decreases proliferation and clonogenic capacity in PTC K1 cells. (**a**) Quantification of K1 cell numbers over a 120 h period. (**b**) Representative images of stained colony-forming units (CFUs). (**c**) Quantification of CFUs following approximately 10 days of colony formation. Data are presented as mean ± SD. Statistical significance was evaluated using an unpaired *t*-test; *n* = 3 per group (no technical replicates), * *p* ≤ 0.05.

**Figure 5:**
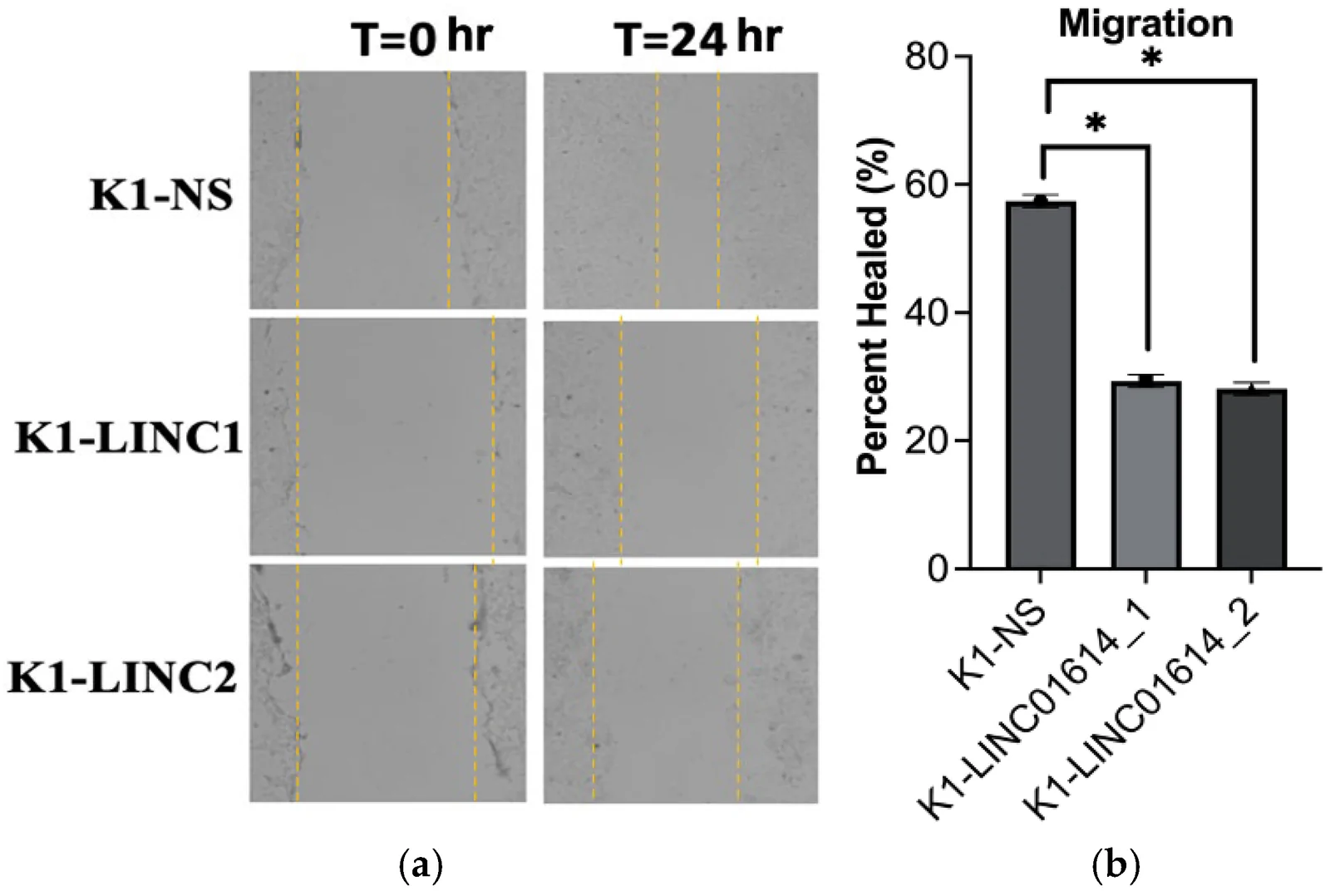
Transcriptional repression of LINC01614 reduces the migratory capacity of PTC K1 cells in vitro. (**a**) Representative brightfield microscopy images of the induced wound field at 0 and 24 h. Dashed lines represent wound field walls for easier viewing. (**b**) Quantification of cell migration based on percent wound healing (%). Statistical significance was assessed using an unpaired *t*-test; *n* = 3 per group (no technical replicates). * *p* ≤ 0.05.

**Figure 6:**
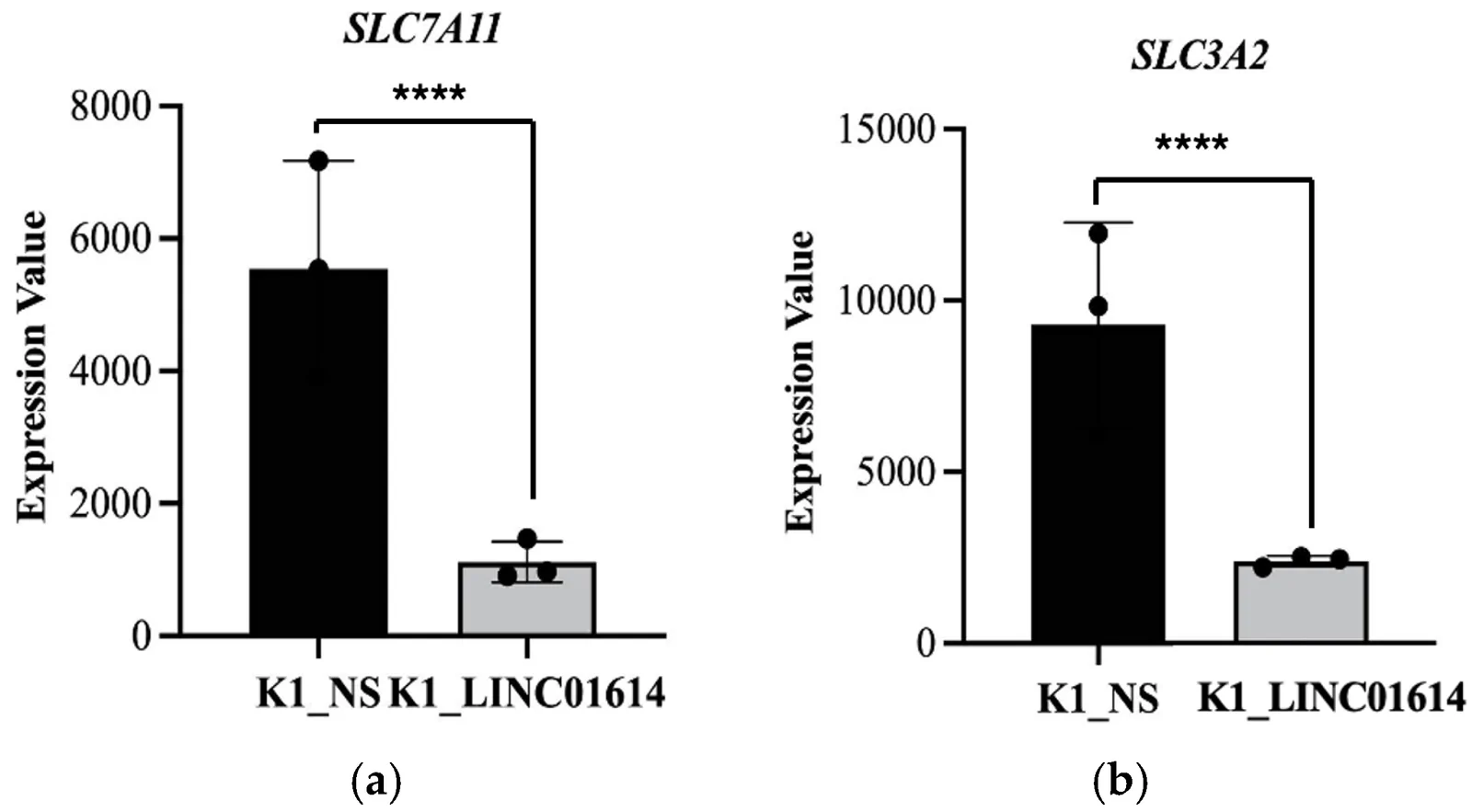
LINC01614 repression reduces expression of the anti-ferroptotic genes SLC7A11 and SLC3A2. RNA-seq analysis demonstrated significantly decreased expression of (**a**) SLC7A11 and (**b**) SLC3A2, which encode components of System xCT, in K1-LINC01614_2 cells relative to K1-NS control cells. Differential expression analysis was performed using DESeq2 with the Wald test and Benjamini–Hochberg correction for multiple comparisons. *n* = 3 per group, **** adjusted *p* ≤ 0.0001.

**Figure 7:**
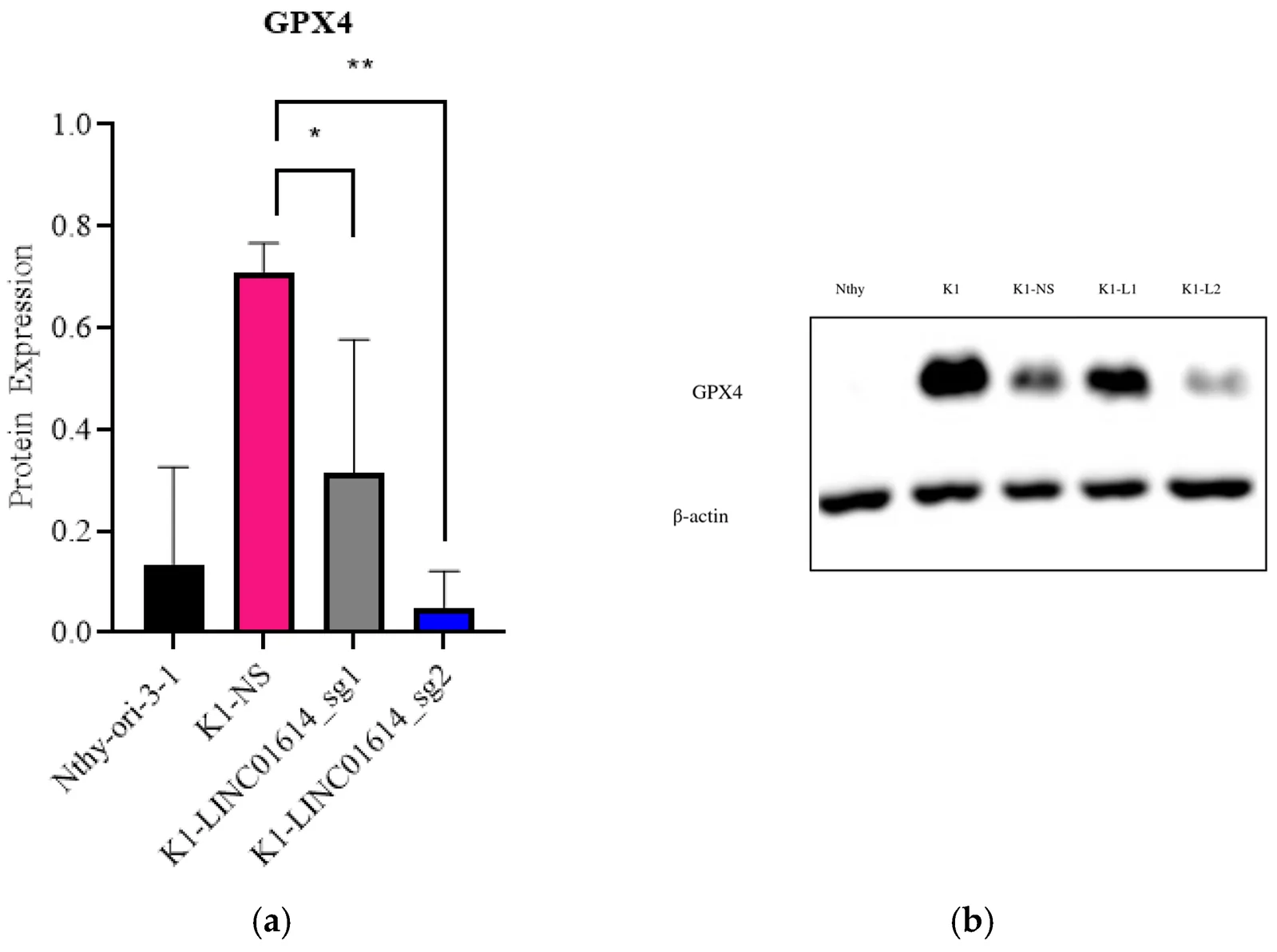
LINC01614 knockdown is associated with reduced GPX4 protein expression. (**a**) Densitometric quantification of GPX4 protein expression normalized to β-actin. (b) Representative Western blot showing GPX4 and β-actin expression. Parental K1 was included as a reference in the representative blot but was not included in the densitometric or statistical analysis. Quantification was performed for Nthy-ori-3-1, K1-NS, K1-LINC01614_1, and K1-LINC01614_2. Statistical comparisons between K1-NS and K1-LINC01614 knockdown cells were conducted using an unpaired two-tailed Student’s *t*-test. Data are presented as mean ± SD; *n* = 2 per group (each sample loaded once), * *p* ≤ 0.05, ** *p* ≤ 0.01.

**Figure 8:**
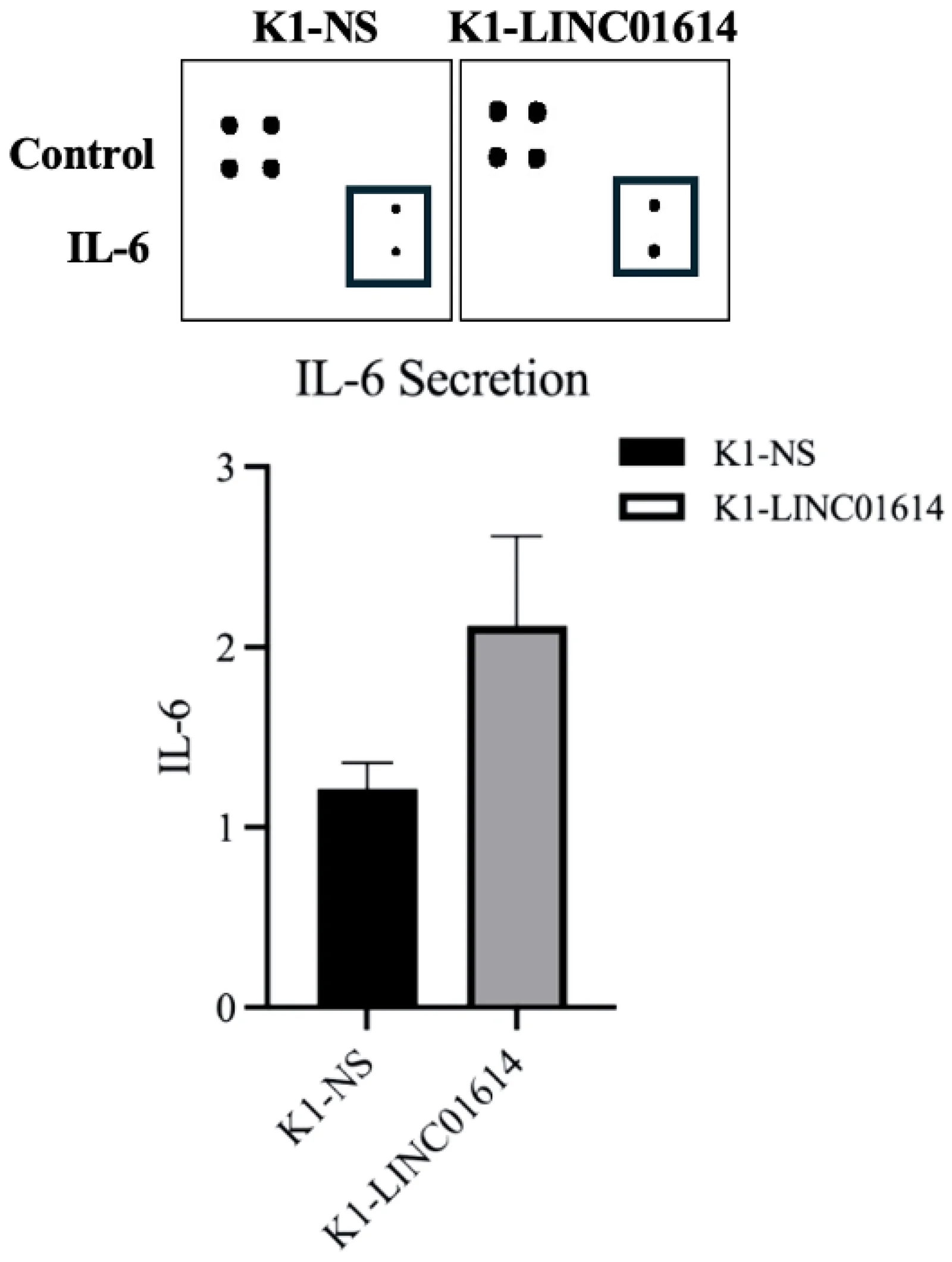
Exploratory cytokine array analysis revealed increased IL-6 signal following LINC01614 repression. Cytokine array results and quantification depicted a 75% increase in IL-6 in the LINC01614 knockdown cell line compared to the control; *n* = 1 per group (3 technical replicate wells per group).

**Figure 9:**
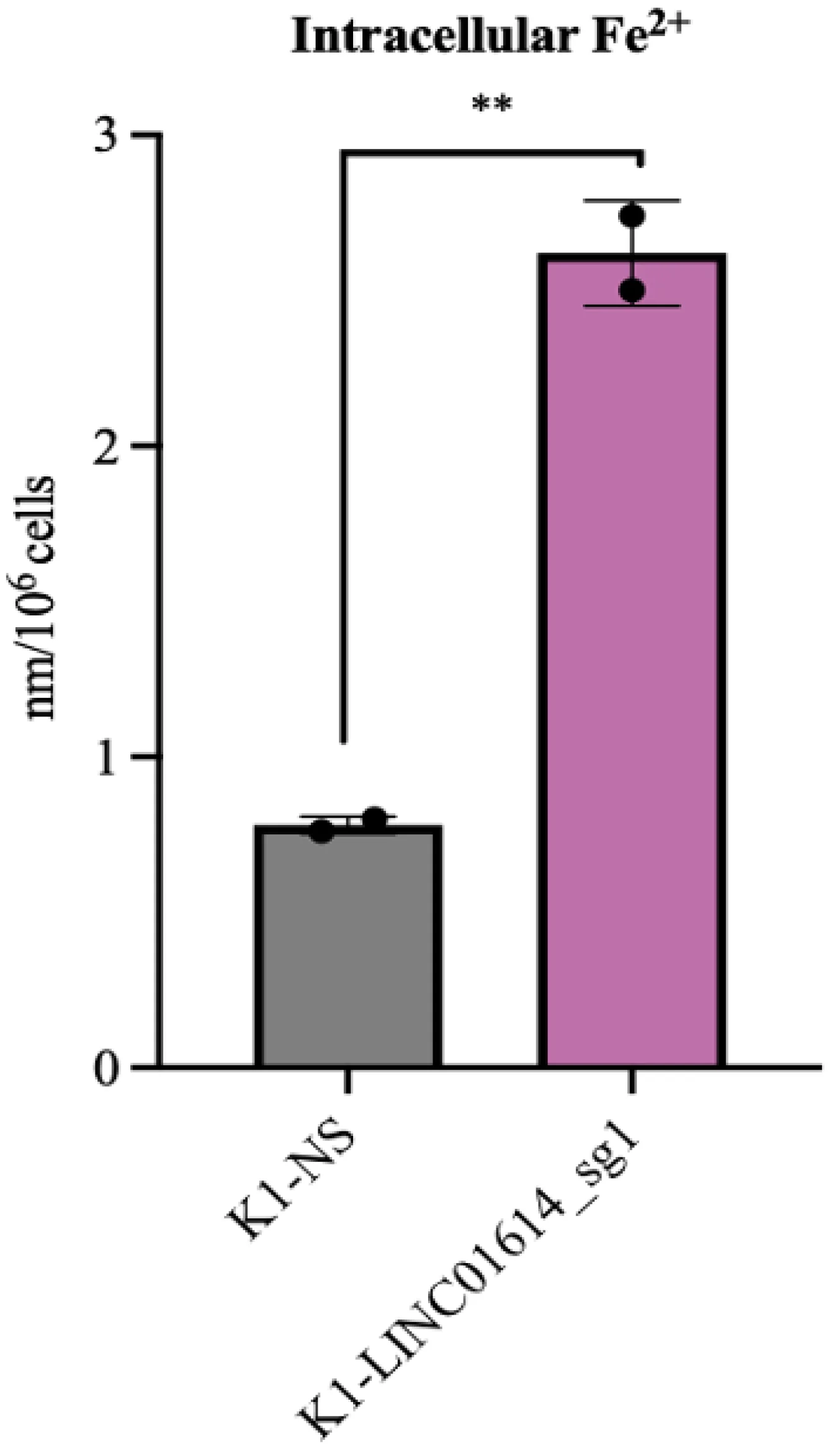
Transcriptional repression of LINC01614 increases intracellular iron levels. Measurement of total intracellular ferrous iron demonstrated an approximately 3.4-fold increase in LINC01614 knockdown cells relative to control cells. Statistical comparisons between groups were conducted using an unpaired two-tailed Student’s *t*-test. Data are presented as mean ± SD; *n* = 2 per group (3 technical replicate wells per experiment), ** *p* ≤ 0.01.

**Figure 10:**
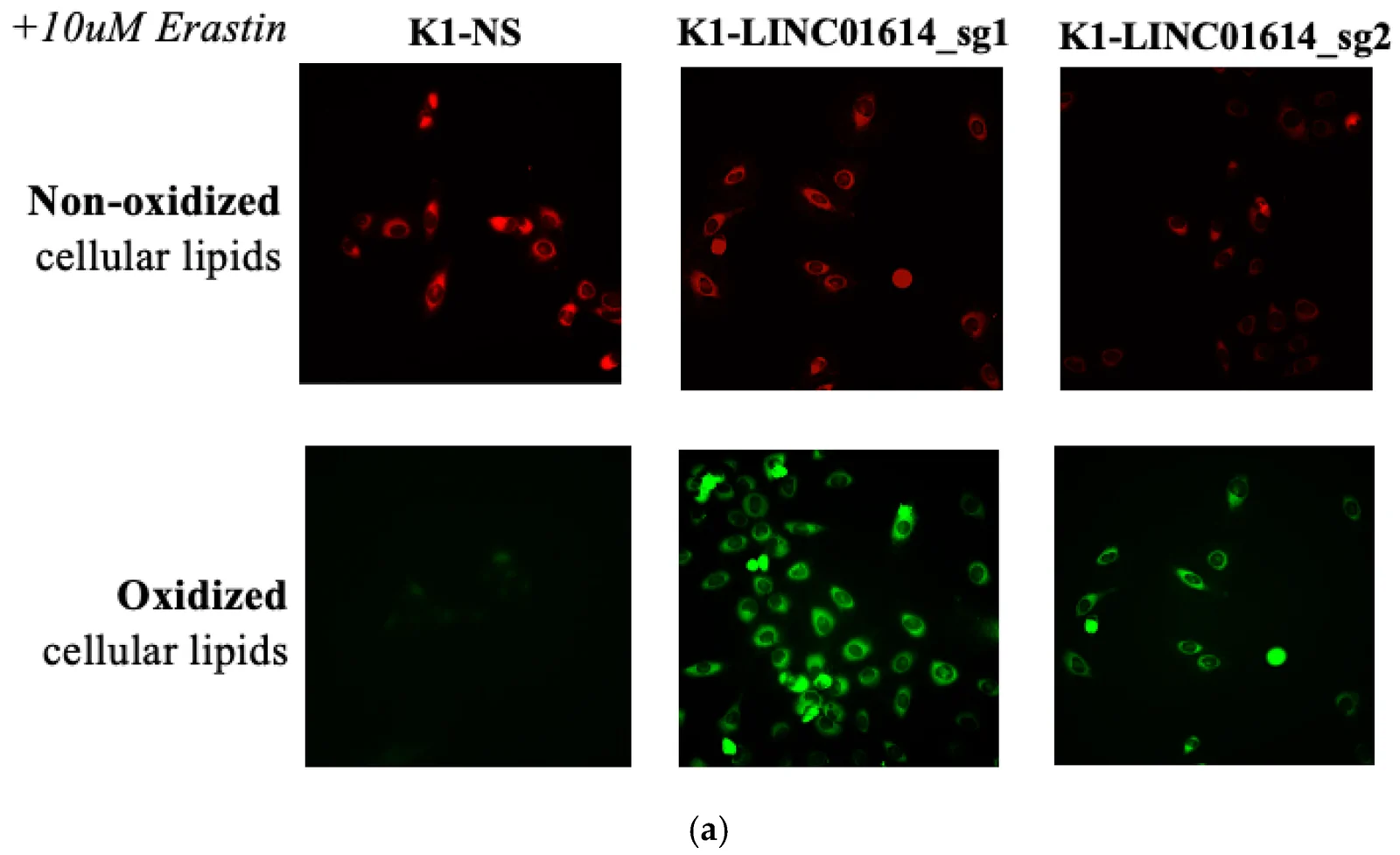

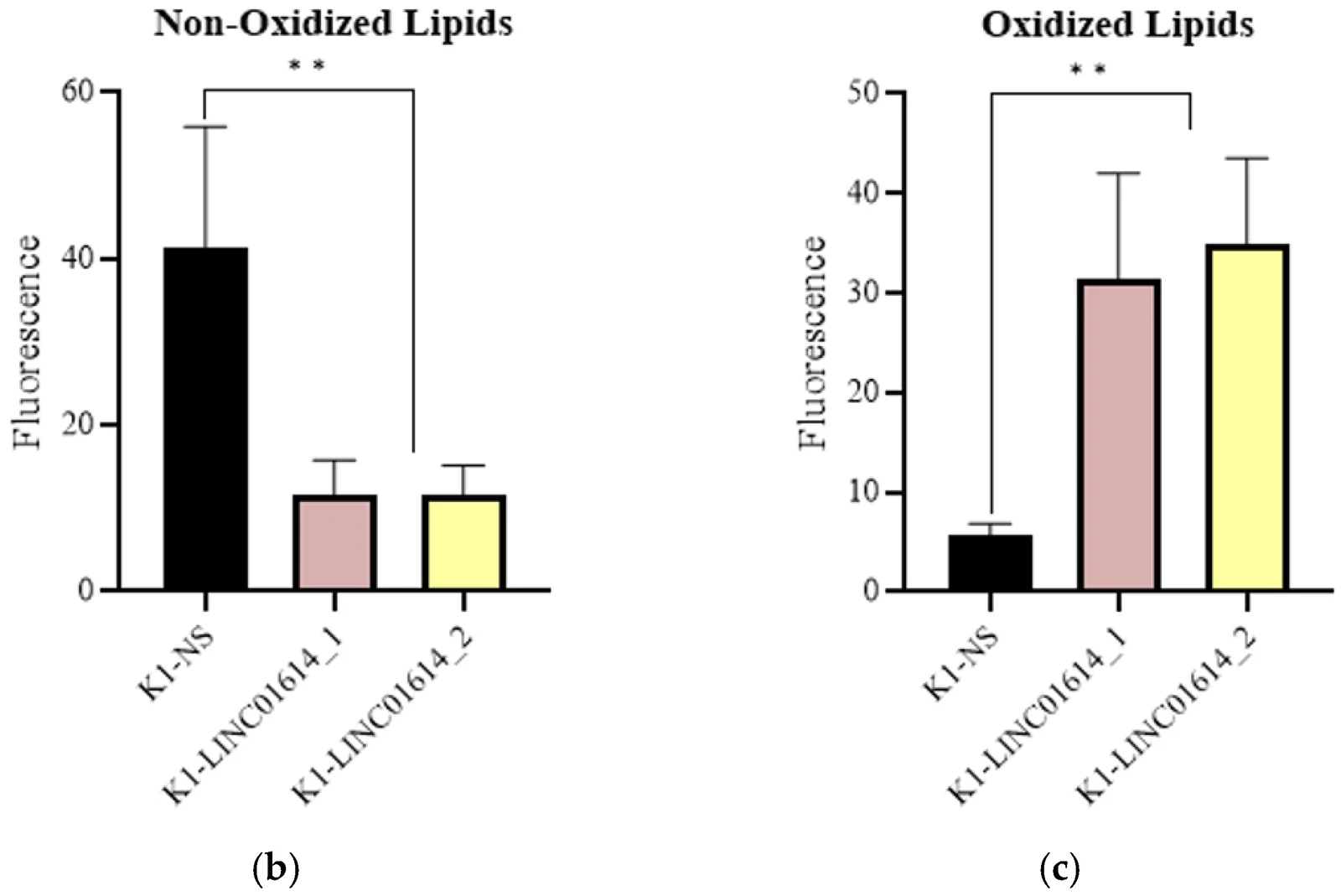
Lipid peroxidation levels were increased when LINC01614 cells were treated with a ferroptosis inducer. (**a**) Fluorescent microscope images depicting differences in non-oxidized (red) and oxidized (green) cellular lipids; (**b**) quantification of non-oxidized lipids; (**c**) quantification of oxidized lipids; Statistical comparisons between groups were conducted using an unpaired two-tailed Student’s *t*-test, *n* = 2 per group (no technical replicates), ** *p* ≤ 0.01.

**Figure 11:**
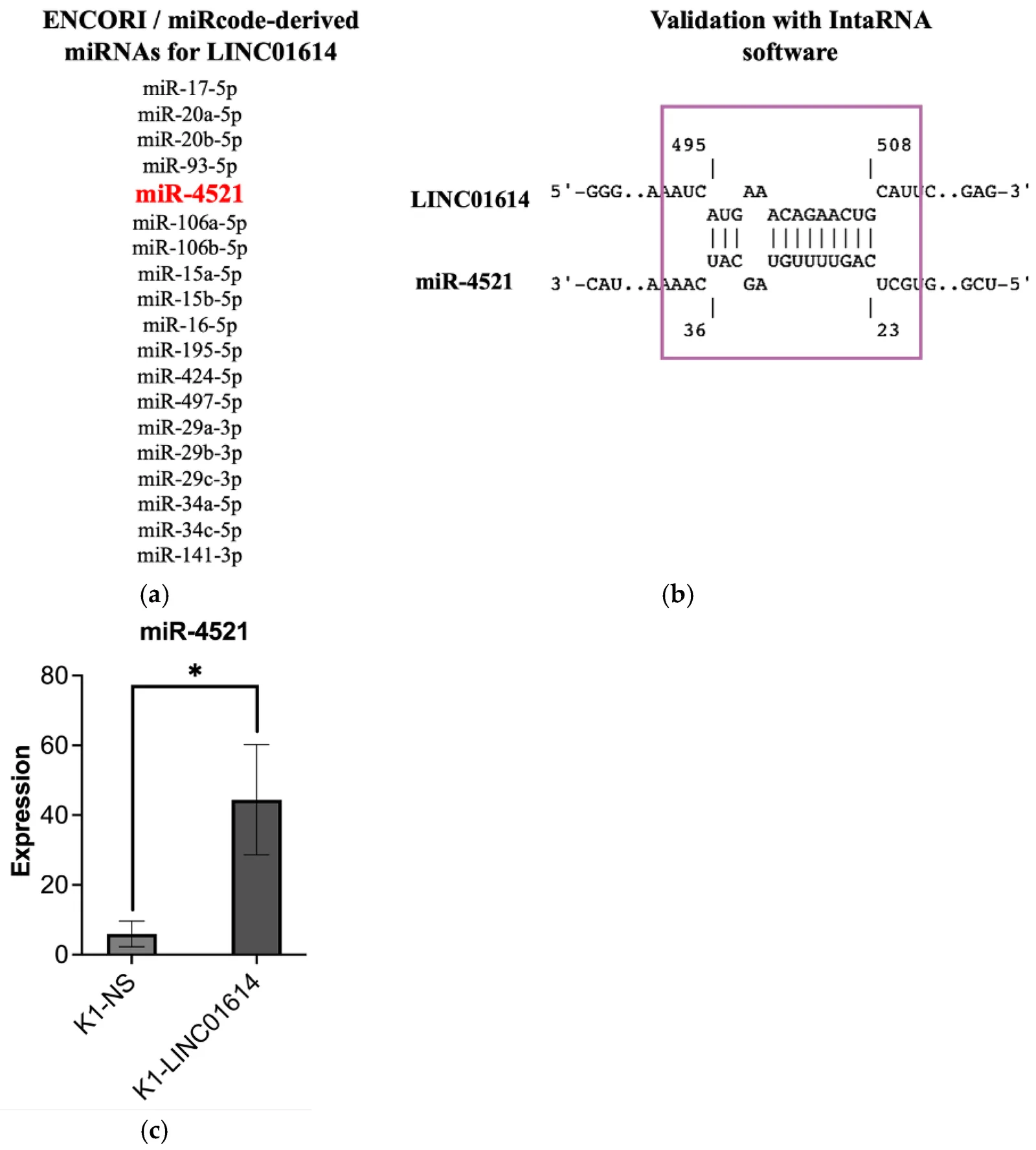
In silico analysis identifies miR-4521 as a predicted miRNA binding partner of LINC01614. (**a**) Nineteen potential miRNA binding partners for LINC01614 identified using ENCORI and miRcode. (**b**) Predicted interaction between LINC01614 and miR-4521 generated using IntaRNA software. (**c**) qRT-PCR analysis of miR-4521 expression in LINC01614 knockdown cells relative to control cells. Data are presented as mean ± SD from three independent experiments. Statistical significance was evaluated using an unpaired two-tailed Student’s *t*-test; *n* = 3 per group (3 technical replicates per experiment), * *p* ≤ 0.05.

**Figure 12:**
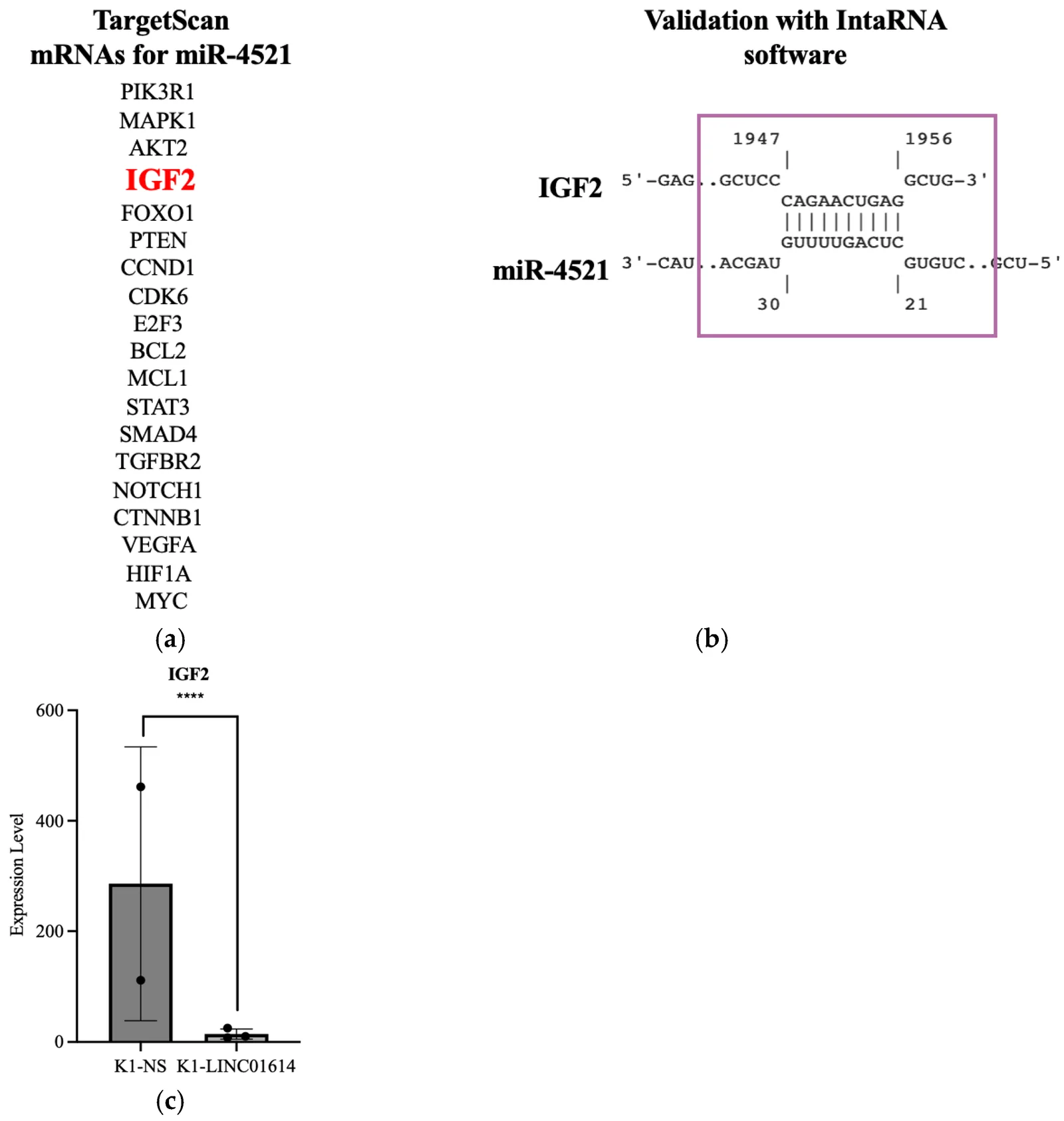
In silico analysis identifies IGF2 as a predicted mRNA target of miR-4521. (**a**) Nineteen potential mRNA binding partners of miR-4521 identified using TargetScan software (v8.0). (**b**) Predicted interaction between miR-4521 and IGF2 generated using IntaRNA software. (**c**) qRT-PCR analysis of IGF2 expression in LINC01614 knockdown cells relative to control cells. Statistical comparisons between groups were performed using an unpaired two-tailed Student’s *t*-test; *n* = 3 per group (3 technical replicates per experiment), **** *p* ≤ 0.0001.

**Figure 13:**
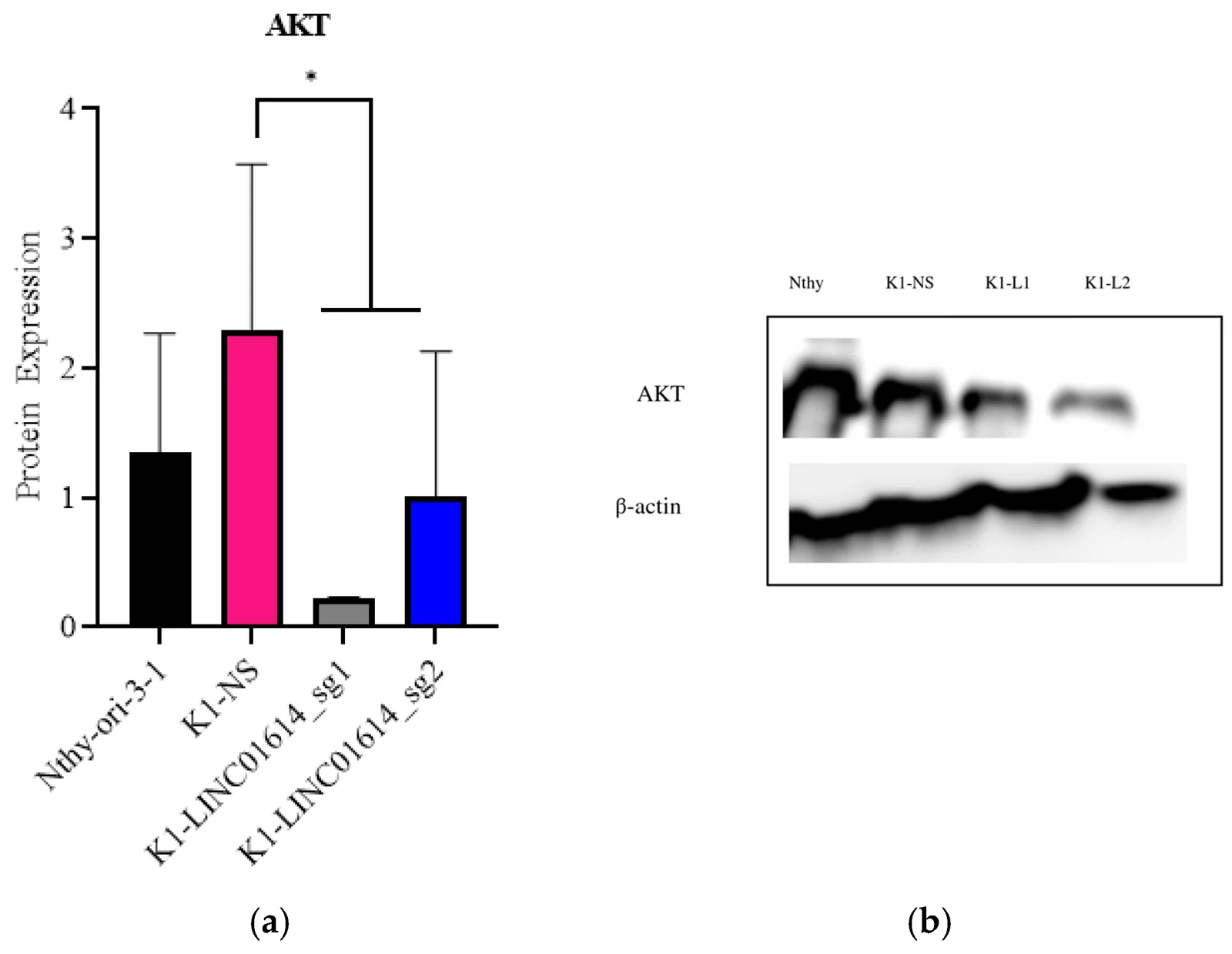
LINC01614 repression is associated with reduced total AKT protein expression. (**a**) Densitometric quantification of AKT protein expression normalized to β-actin. (**b**) Representative Western blot showing AKT and β-actin expression in Nthy-ori-3-1, K1-NS, K1-LINC01614_1, and K1-LINC01614_2 cells. Statistical comparisons between K1-NS and the K1-LINC01614 knockdown cells were performed using an unpaired two-tailed Student’s t-test. Data are presented as mean ± SD; *n* = 2 per group (each sample loaded once), * *p* ≤ 0.05.

**Figure 14:**
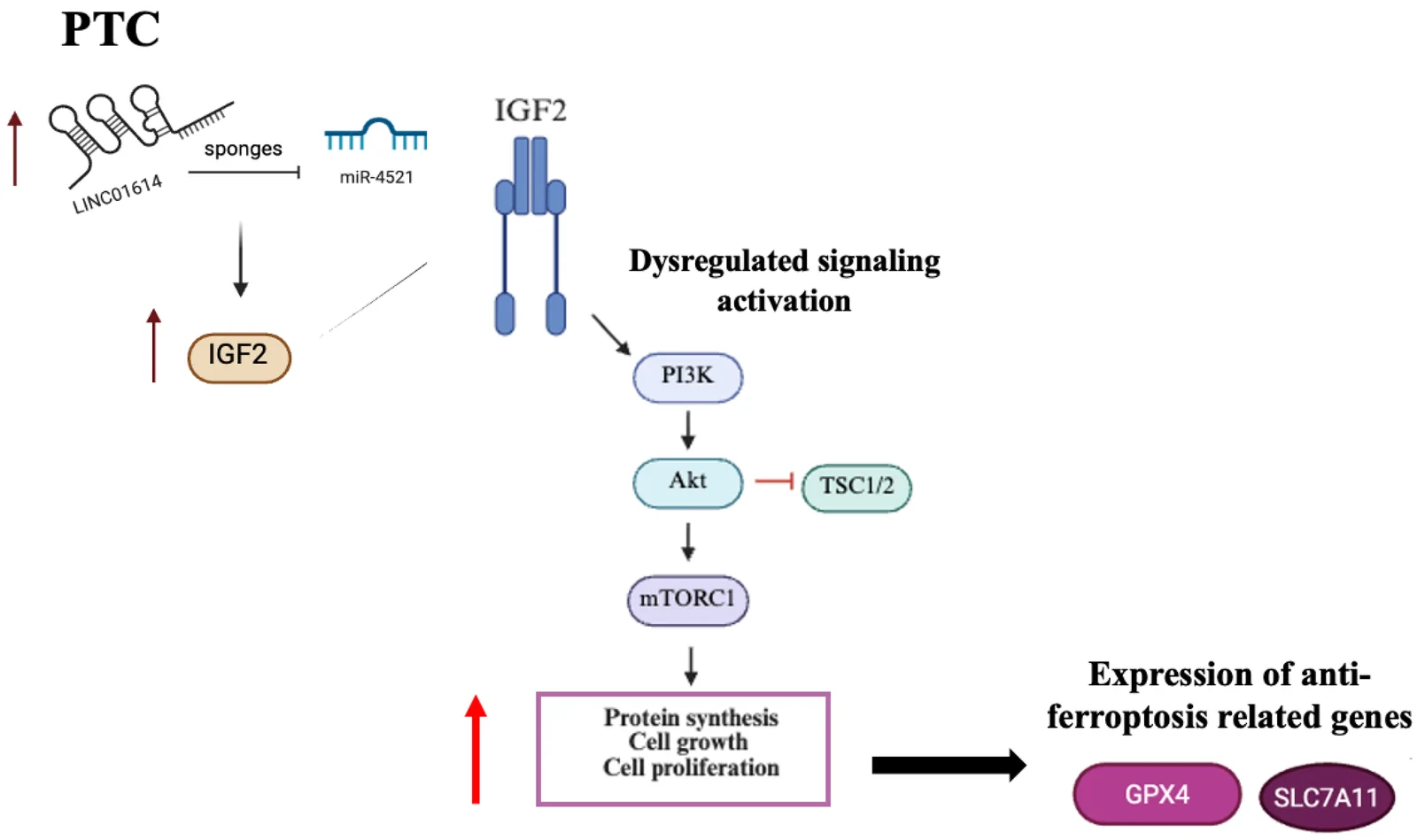
ceRNA axis for LINC01614-miR-4521-IGF2 in PTC. Schematic representing upregulated expression of LINC01614 leading to a sponging of miR-4521 and overexpression of IGF2. Overexpression of IGF2 leads to increased PI3K/Akt signaling and thus increased protein synthesis and cell growth (red arrow) as well as expression of anti-ferroptotic genes.

## Data Availability

The original contributions presented in this study are included in the article. Further inquiries can be directed to the corresponding authors.

## Abbreviations

The following abbreviations are used in this manuscript:

AKT	Protein Kinase B
BODIPY	Boron-Dipyrromethene Lipid Peroxidation Probe
CFU	Colony-forming unit
CRISPRi	CRISPR interference
DEGs	Differentially expressed genes
ENCORI	Encyclopedia of RNA Interactomes
GPX4	Glutathione Peroxidase 4
IGF2	Insulin-like Growth Factor 2
IL-6	Interleukin 6
LB	Luria broth
lncRNA	Long non-coding RNA
LINC01614	Long intergenic non-coding RNA 01614
MAPK	Mitogen-Activated Protein Kinase
NCBI	National Center for Biotechnology Information
NEB	New England Biolabs
PI3K	Phosphoinositide 3-Kinase
PTC	Papillary thyroid cancer
ROS	Reactive oxygen species
RSEM	RNA-seq by Expectation Maximization
RQ	Relative quantification
sgRNA	Single guide RNA
SLC3A2	Solute Carrier Family 3 Member 2
SLC7A11	Solute Carrier Family 7 Member 11
STAR	Spliced Transcripts Alignment to a Reference
xCT	Cysteine/glutamate antiporter

## Appendix A

**Table A1:** Selected differentially expressed genes associated with ferroptosis, oxidative stress, metabolism, and tumor-associated signaling following LINC01614 repression. Differentially expressed genes (DEGs) identified by RNA sequencing of K1-LINC01614_2 and K1-NS cells are shown with corresponding log_2_ fold-change values, adjusted *p*-values (FDR), direction of regulation, and relevant biological functions or pathways. DEGs were defined as genes with an adjusted *p*-value < 0.05 and an absolute log_2_ fold change ≥ 1.

Gene	Log_2_ Fold Change	Adjusted *p*-Value	Regulation	Relevant Function
AIFM2 (FSP1)	−4.14	0.0085	Downregulated	Ferroptosis suppression/lipid ROS defense
SESN2	−2.01	9.59 × 10^−5^	Downregulated	Oxidative stress/NRF2 signaling
CHAC1	−2.73	1.64 × 10^−15^	Downregulated	Glutathione metabolism/stress response
HERPUD1	−1.47	4.48 × 10^−5^	Downregulated	ER stress response
ATF6	−1.33	0.0239	Downregulated	Unfolded protein response
PLIN2	−6.10	9.59 × 10^−5^	Downregulated	Lipid storage and metabolism
SLC31A1	−1.35	4.23 × 10^−5^	Downregulated	Metal ion transport
GOT1	−1.64	3.72 × 10^−4^	Downregulated	Cellular metabolism/redox balance
SLC7A11	−2.31	9.56 × 10^−9^	Downregulated	System xCT/cystine transport
CTH	−3.19	2.41 × 10^−13^	Downregulated	Cysteine biosynthesis
SLC1A5	−1.47	2.71 × 10^−5^	Downregulated	Glutamine transport
SLC38A1	−2.38	6.40 × 10^−7^	Downregulated	Amino acid transport
SLC38A2	−1.11	2.10 × 10^−4^	Downregulated	Amino acid transport
SLC7A5	−3.65	9.32 × 10^−9^	Downregulated	LAT1 amino acid transport
IGFBP2	+2.46	0.0274	Upregulated	IGF-associated signaling
EFNB2	+1.19	0.0019	Upregulated	Cell signaling/migration
RGCC	+1.98	0.0217	Upregulated	Cell-cycle regulation
